# The impact of perceived emotions on toddlers' word learning

**DOI:** 10.1111/cdev.13799

**Published:** 2022-05-30

**Authors:** Lizhi Ma, Katherine Twomey, Gert Westermann

**Affiliations:** ^1^ Department of Psychology Lancaster University Lancaster UK; ^2^ Institute of Advanced Technology Westlake Institute for Advance Study Hangzhou China; ^3^ School of Engineering Westlake University Hangzhou China; ^4^ Division of Human Communication, Development and Hearing University of Manchester Manchester UK

## Abstract

Others' emotional expressions affect individuals' attention allocation in social interactions, which are integral to the process of word learning. However, the impact of perceived emotions on word learning is not well understood. Two eye‐tracking experiments investigated 78 British toddlers' (37 girls) of 29‐ to 31‐month‐old retention of novel label‐object and emotion‐object associations after hearing labels presented in neutral, positive, and negative affect in a referent selection task. Overall, toddlers learned novel label‐object associations regardless of the affect associated with objects but showed an attentional bias toward negative objects especially when emotional cues were presented (*d* = 0.95), suggesting that identifying the referent to a label is a competitive process between retrieval of the learned label‐object association and the emotional valence of distractors.

**Abbreviations**
AOI: areas of interest
BF: Bayes factor
ERP: event‐related potential
HSD: Honest Significant Difference
LMEM: linear mixed‐effect model
Nc: negative component
Oxford CDI: Oxford Communicative Development Inventory
RT: retention

## INTRODUCTION

In social contexts, children need to integrate and evaluate the diverse information they perceive to learn word‐world associations such as linguistic, attentional, and social cues (Hollich et al., 2000; Monaghan et al., 2018; Yu & Ballard, 2007). From discriminating linguistic speech from nonlinguistic speech as neonates (Eimas, 1971; Vouloumanos & Werker, 2007) to speaking their first meaningful word at around 12 months (Benedict, 1979), infants absorb phonological patterns they hear based on the statistical regularities in linguistic input. They acquire the most frequently heard nouns, verbs, and adjectives and extend the range of those words (Clark, 1995; Saffran et al., 1996). In doing so, infants attend to language that is directed to them, to the objects that are physically and conversationally present, and to social pragmatic cues that attract joint attention from both their caregivers and them (Clark, 2016; Tomasello, 2003). Thus, the cues perceived by children influence their learning of word‐world associations.

Meanwhile, the roles of different situational factors in word learning have been explored widely. For instance, in tasks where toddlers need to identify the referent for a word among a set of competitors, learning outcomes are better when 36‐month‐olds encounter repetitive competitors than constantly varying ones (Axelsson & Horst, 2014). Similarly, the 30‐month‐olds recognize new‐learned label‐object associations better at test when they encounter fewer competitors in learning (Horst et al., 2010); and 24‐month‐olds' recognition is improved after learning label‐object associations when objects are presented on variable rather than on constant backgrounds (Twomey et al., 2017). Thus, children can capitalize on information they perceive from a range of modalities when they are learning new words. Importantly, word learning takes place in a social context where emotional information is a common social pragmatic cue and affects infants' attention (e.g., Quinn et al., 2020), behaviors (e.g., Moses et al., 2001), as well as learning (Singh et al., 2004). However, how emotional expressions perceived by children affect early word learning remains unclear.

Even neonates react to emotional information, preferring to look at stimuli accompanied by infant‐directed speech, which features an affectively positive prosody, over stimuli accompanied by adult‐directed speech (Cooper & Aslin, 1990). Moreover, happy prosody elicits neural responses distinct to responses to fear, anger, and neutral prosody in newborns (Zhang et al., 2019). Then, from 6–7 months, infants gradually attend more to negative expressions than to positive ones (Hoehl, 2014; Vaish et al., 2008). For example, 7‐month‐old infants look longer to fearful than to happy faces in a visual preference task (Kotsoni et al., 2001; Nelson & Dolgin, 1985). On the neural level, event‐related potential (ERP) studies reliably report that negative emotional expressions evoke a larger negative component (Nc), which reflects attentional processes, relative to happy and neutral expression in infants older than 7 months (e.g., fearful vs. neutral and fearful vs. happy facial expressions: Hoehl & Striano, 2010; Leppänen et al., 2007). Overall, infants start by allocating more visual attention to positive emotional expressions and increase their attention to negative emotional expressions as they develop.

In addition to face processing, perceived emotions also affect infants' attention allocation in object processing tasks. Flom and Johnson (2011) reported that after habituating to an actress directing happy and disgusted expressions toward two novel objects, 12‐month‐old infants looked longer at the object paired with happy expression in a preferential looking task, both after a 5‐min delay and also the day following habituation. In contrast, Carver and Vaccaro (2007) demonstrated that 12‐month‐old infants showed an enhanced ERP response to negatively conditioned objects. In this study, infants observed their caregivers interacting with three objects in an emotionally positive (happy), negative (disgusted), and neutral manner, respectively. After a 20‐min delay, infants' ERP responses to the objects were measured. A larger Nc was found when infants saw the object associated with the negative emotion compared to the objects associated with positive or neutral emotions. A similar effect was also found in 6‐month‐old infants, who showed a stronger Nc for objects which they had observed alongside a fearful face compared to a neutral face (Hoehl & Striano, 2010). Overall, infants' neural responses are enhanced when processing negatively conditioned objects, but their looking preference is toward positively conditioned objects.

Further, emotional vocalizations influence infants' processing of linguistic information. Singh et al. (2004) investigated the effect of emotional vocalization on infants' word recognition. Seven‐month‐old infants recognized positively familiarized words in positively spoken fluent speech but not in neutral speech; they also recognized neutrally familiarized words in neutral speech but not in positive speech. In contrast, 10‐month‐old infants recognized positively familiarized words in both positive and neutral speech, but not neutrally familiarized words in positive speech. Singh et al. accounted for this finding by positing an attention bias toward positively spoken words. Specifically, they suggested that greater attention to positive vocalizations may have led to deeper encoding during familiarization and thus facilitated infants' generalization of the positively spoken word to different contexts.

Based on the above findings, emotionally conditioned stimuli (e.g., objects, spoken words) are allocated more attention than neutral stimuli, which may promote encoding and influence learning outcomes. Specifically, differences in attention allocation due to differences in perceived emotions may lead to differences in the processing of visual and vocal information. Therefore, perceived emotional information should affect attention allocation during word learning. Despite little research investigating how these perceived emotions might affect children's word learning outcomes, two studies with adults have served to shed some light on the learning of pseudowords associated with emotions (Fritsch & Kuchinke, 2013; Kuchinke et al., 2015). In these studies, over a period of 5 days, participants learned the association between written pseudowords and emotionally neutral, positive, and negative pictures. They were then tested in a lexical decision and an ERP task to measure retention and neural processing of the pseudowords and a further task to rate the emotionality of the pseudowords. Although words associated with positive and negative pictures were rated higher on emotionality and elicited greater neural responses compared to neutral words, retention was similar for all words, suggesting that emotions associated with novel words have no effect on the learning of these words but induced enhanced attention on the neural level (Fritsch & Kuchinke, 2013; Kuchinke et al., 2015).

Interestingly, as symbolic units which bear no emotional meanings per se, pseudowords associated with emotions evoke an attentional bias in adults (Fritsch & Kuchinke, 2013; Kuchinke et al., 2015). For young word learners, when they learn label‐object associations in social interactions full of emotional expressions (Clark, 2016; Fernald et al., 1989), they must process emotions (conveyed through tone of voice, facial expression, and body posture and gestures), language, and objects simultaneously and associate these different cues from the outset, which raises the possibility that their attention during learning and recognition is affected by the associations being formed (e.g., Hoehl & Striano, 2010). Thus, to fill this gap in our understanding of the early learning of label‐object associations, this study focused on the impact of perceived emotions on children's learning and retention of label‐object associations.

To integrate cues of perceived emotions, language, and objects in a word learning task, the experimental design should embed external emotional information in the process of learning novel label‐object associations but should separate the impact of perceived emotions and newly learned labels on the retrieval of label‐object associations when recall is tested. These requirements can be fulfilled by the *referent selection* and *retention* paradigm (e.g., Axelsson & Horst, 2014; Twomey et al., 2017). In the referent selection phase, a set of objects is presented side‐by‐side live or on a screen, typically consisting of two familiar and one novel object. A novel label is then given, for example, “Look at the *toma*,” with the expectation that infants should select the novel object as the referent. Typically, several novel label‐object pairs (3–4) are taught in this manner in a single session. In the retention test, the novel objects learned in the referent selection phase are then presented side‐by‐side to children, and they are asked to get, or look at, one of the objects by the label attached to it.

In a departure from the typical procedure in referent selection tasks (e.g., Hilton et al., 2019; Twomey et al., 2017), which seeks to minimize social cues, in this study, an actress also appeared on the screen and labeled the objects using neutral, positive (happy), or negative (disgusted) affect. Afterward, we tested short‐term (after 5 min) and long‐term (after 1 day) retention by measuring both toddlers' looking preference and pointing responses to target objects. To tease apart the roles of perceived emotions and language in retrieval of newly learned associations, we included both label and no‐label trials in the retention phases. The label trials examined toddlers' retention of novel label‐object associations, while the no‐label trials tested whether toddlers had associated objects with the affect encountered during referent selection.

Thus, we presented 30‐month‐old toddlers with this word learning paradigm. In contrast to 24‐month‐olds, who fail to retain any novel label‐object associations in either in‐person or screen‐based tasks after learning in a typical referent selection task (e.g., Hilton et al., 2019; Horst & Samuelson, 2008), 30‐month‐olds have been shown to retain the novel associations (Horst et al., 2010). Further, since 36‐month‐olds robustly retain novel associations even in a challenging referent selection task with novel competitors (Axelsson & Horst, 2014), we selected 30‐month‐olds with the aim that they should demonstrate variation in the retention of novel label‐object associations in the current design, thus avoiding ceiling or floor effects.

Based on previous findings that infants over 7 months old allocate more attention to objects associated with negative affect (Hoehl, 2014) and better generalize positive vocalizations compared with neutral affect (Singh et al., 2004), we hypothesized that, compared to the novel label‐object associations delivered in neutral affect, toddlers would better retain the labels delivered with positive and, particularly, negative affect, indicated by greater proportion looking or more pointing to targets in the retention phases. We also expected toddlers to associate positive and negative affect with the corresponding objects during retention tests.

## EXPERIMENT 1

### Method

#### Participants

Thirty‐eight 29‐ to 31‐month‐old typically developing, white British monolingual English‐learning toddlers from middle‐class backgrounds living in the Lancashire area, United Kingdom, participated (21 girls, *M* = 30.52 months, *SD* = 0.81 months). An additional eight toddlers were excluded from analyses for failure to remain on the caregiver's lap (4); caregiver intervention (2); poor calibration (1); and experimenter error (1). All toddlers were recruited from Lancaster Babylab's database of caregivers who had expressed an interest in taking part in developmental research. Toddlers' mean productive vocabulary was 372.20 (*SD* = 68.17, range = 92–418, the 50th, 75th, and 90th percentiles are 397, 410, and 415.20, respectively), as measured by parental completion of the Oxford Communicative Development Inventory (Oxford CDI) and is above the 75th percentile of normed data (Hamilton et al., 2000). Caregivers gave consent to participate and received travel reimbursement. Toddlers were given a story book for taking part. The research project was granted ethics approval by the Lancaster University Research Ethics Committee.

#### Stimuli and design

**FIGURE 1:**
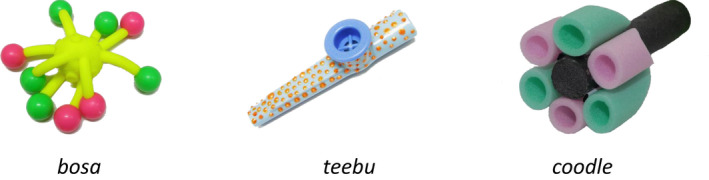
Novel objects and labels

**FIGURE 2:**
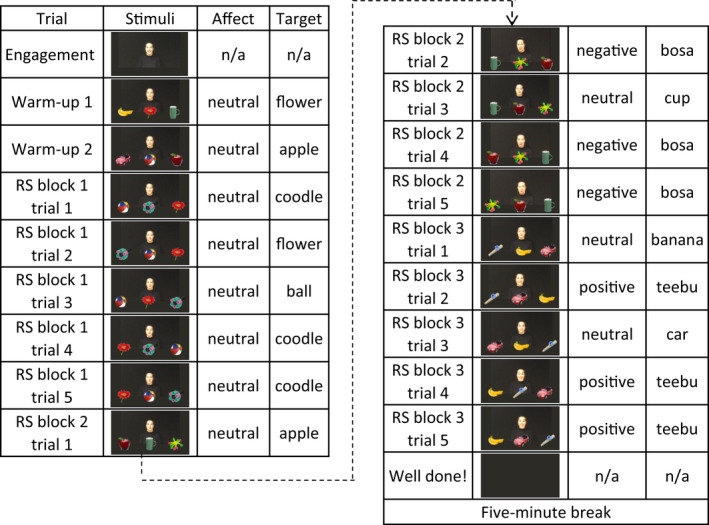
An example of warm‐up and referent selection (RS) phase. For a video example of the referent selection phases (see https://doi.org/10.17605/OSF.IO/72KPS)

**FIGURE 3:**
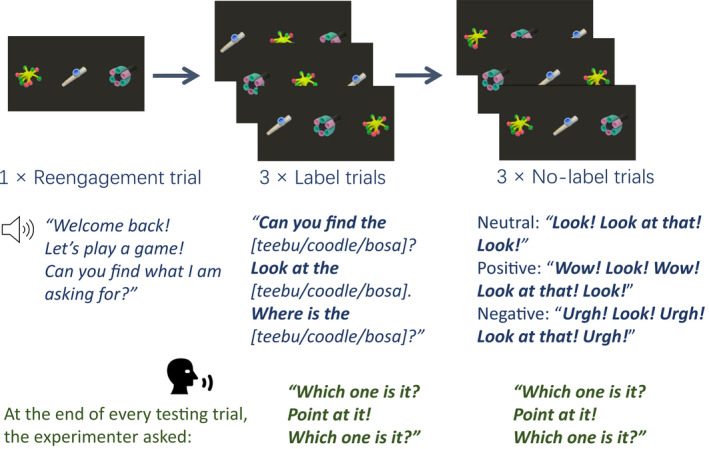
An example of retention trials in Experiment 1. For a video example of the retention phase (see https://doi.org/10.17605/OSF.IO/72KPS)

Six known objects were selected because their labels are familiar to 2‐year‐old toddlers (Fenson et al., 1994). Known objects consisted of photographic images of an apple, a ball, a banana, a car, a cup, and a flower. Three novel objects (see Figure 1) and three two‐syllable nonwords (*coodle*, *bosa*, and *teebu*) were selected from the NOUN online database of objects and labels were unfamiliar to toddlers of this age (Horst & Hout, 2016). All objects were approximately the similar size (Table S1). Video recordings of an actress labeling the objects were recorded on an iPhone SE, which was found to provide higher quality recordings than a dedicated video camera. Video stimuli combining the video recordings and the objects were generated in Microsoft PowerPoint 2016 and converted into .wmv format. Video stimuli were displayed on a dark gray background (R = 45, G = 43, and B = 37) and consisted of five types of videos: engagement, warm‐up, referent selection, reengagement, and retention (see Figures 2 and 3; see Supporting Information for a detailed description of video stimuli; video examples: https://doi.org/10.17605/OSF.IO/72KPS).

##### Time course of stimulus presentation

For the warm‐up and referent selection phases, the detailed time course and label onsets of stimuli are presented in Supporting Information (Table S1). Retention trials lasted 8500 ms. In label trials, first label onset was at 2000 ms, second label onset at 4000 ms, and third label onset at 6000 ms after trial onset. For no‐label trials, the onsets of emotional cues were at 2000 ms, 4000 ms, and 6000–6200 ms (Neutral: *Look*! Positive: *Wow*! Negative: *Urgh*!) after trial onset. After every testing trial, the three novel objects remained on the screen and the experimenter encouraged toddlers to point at the target.

##### Stimulus ratings

In order to ensure that the effect of our stimuli was perceived as we intended, we asked 60 adult participants (46 females, *M* = 20.9 years old, *SD* = 7.21) to complete an online survey to assess the valence of the actress's emotional expressions and voice (neutral, positive, and negative). Participants watched the 27 referent selection videos and listened to the six retention audio recordings, rating each stimulus as either neutral, positive, or negative, that is, *Please watch this video and assess the speaker's emotion* with options of *Neutral*, *Positive*, and *Negative*. We calculated average percentage agreement; agreement of 100% represents complete agreement and 33.33% indicates that participants chose three options randomly. For the referent selection video ratings, agreement on the neutral videos was 95.56%, the positive videos 100%, and the negative videos 98.68%. The retention label trials, in which audio was presented in neutral affect, had a rating of neutral at 92.6%. The retention no‐label trials, which presented three affective cues, had a rating of neutral at 96.61%, and of positive and negative each at 100%.

#### Procedure

The procedure of the experiment ran as follows: first, toddlers learned three novel label‐object associations in the referent selection phase; then, they took a 5‐min break before the first retention phase (RT1). Once the child had completed referent selection and RT1, they were invited to return for the second retention phase (RT2) within 36 h of the first day's testing. On arrival of the participants, before the experiment began, the experimenter introduced caregivers to the procedure and showed them pictures of the objects used in the study to confirm that the participants knew the names of the six known objects but did not know the three novel objects. All caregivers reported that participants were familiar with the known objects and unfamiliar with the novel objects. Caregivers were also asked to complete the Oxford CDI (Hamilton et al., 2000). After the experimenter had obtained consent, caregivers and toddlers were guided to a quiet, dimly lit room where participants sat on their caregiver's lap 50–70 cm in front of a 21.5 in. 1920 × 1080 computer screen. A Tobii X120 eye tracker (60 Hz) beneath the screen recorded the child's gaze location, and a video camera above the screen recorded the caregiver and child throughout the study. Caregivers were instructed to turn their head to one side or close their eyes and not to interact with their child. The experimenter monitored caregivers via the live recording by the video camera, and caregiver intervention was coded if they opened their eyes or turned their head to the screen.

Before the referent selection phase, toddlers were presented with a five‐point calibration sequence. The calibration was run until at least four points were calibrated for each eye or up to three times if calibration of both eyes kept failing. Each child was then presented with the engagement trial followed by the two warm‐up trials and the 15 referent selection trials during which the actress and three objects (one novel and two known) were presented in each trial. The actress labeled the three novel objects in neutral, positive, and negative affect, respectively. After the referent selection phase, toddlers took a 5‐min break during which they played with toys (e.g., a ball, blocks) on a play mat. After the break, toddlers returned to their caregiver's lap, and the first retention phase (RT1) began. During the retention phases, in each trial the previously seen three novel objects were presented on the screen, and toddlers were prompted to look at the target object referenced with labels in the label trial block and with affective cues in the no‐label trial block. After each retention trial, the researcher encouraged all participants to point at the target objects by asking *Which one is it? Point at it! Which one is it?* The researcher was behind a curtain and therefore not visible to toddlers during this process. The next retention trial was played when participants had made their choice or had not offered a response after being asked three times. Toddlers' points were recorded by the video camera above the screen for offline coding. The second retention phase (RT2), on the next day, proceeded in an identical manner to RT1 except that the order of the label and no‐label blocks was reversed; that is, toddlers who encountered the label block first in RT1 encountered the no‐label block first in RT2 (see Supporting Information for a detailed description of experimental trials).

#### Data cleaning and model selection

Raw looking time data were exported from Tobii Studio (version 3.2). Areas of interest (AOIs) were defined as rectangles of 536 pixels wide by 424 pixels tall centered on each object's position on the screen. A further face AOI was 471 pixels wide by 419 pixels tall and centered on the actress's face for the referent selection phase. Only gaze points that fell into AOIs entered analyses. Data cleaning and analysis were carried out in the R package *eyetrackingR* (Dink & Ferguson, 2015). Trials in which the eye tracker lost the eyes for more than 50% of the trial duration were excluded from analyses. Thus, 239 out of 297 trials were included for the analyses of the referent selection phase (80.47%); 129 out of 180 trials for RT1 (71.67%) and 117 out of 174 trials for RT2 (67.24%). One participant did not return for RT2; the analyses of RT2 therefore contain six fewer trials than RT1. Toddlers' pointing, specifically index finger pointing, was coded offline by the experimenter; a point was coded as the first point after the third label or cue onset in the retention trials. Points were coded for location (left, middle, and right). Points from 32 toddlers who pointed in more than two trials in each retention phase entered analyses (319 trials). A second coder, naïve to the experimental hypotheses, additionally coded 50% of the recordings, seeing only toddlers' pointing gesture without viewing any stimuli. Thus, the coders were blind to target location. Intercoder reliability was high (Cohen's *κ*: .87).

Data were analyzed in *RStudio* (version 1.0.153; RStudio Team, 2015). We used the *lmer* function from the *lme4* package to fit linear mixed‐effect models (LMEMs) in R (Bates et al., 2015). The effect size reported for LMEMs was $R_{m}^{2}$ and $R_{c}^{2}$, which were the effect sizes explained by fixed effects in the model and by the entire model, respectively (Nakagawa & Schielzeth, 2013). Random effect structure in LMEMs was determined by chi‐squared tests, which were conducted by the *anova* function from the *states* package in the R (Chambers & Hastie, 1992). To take into account individual differences in emotion perception (Lee et al., 2012), we first fit a model with by‐item, by‐participant, by‐affect random intercepts, and by‐participant random slopes for affect. To determine our final random effect structure, we removed random slopes and intercepts in a hierarchical manner. If dropping a random effect improved model fit, this effect was eliminated from the model; if dropping a random effect did not improve model fit, only the random intercepts for items and participants entered the final model. Additionally, multiple comparisons were conducted using post hoc Tukey's Honest Significant Difference (Tukey's HSD) method and reported with adjusted *p*‐values, using the *glht* function from the *multcomp* package (Bretz et al., 2010).

We also report Bayes factors (BF_01_) for focal analyses to provide evidence for the degree to which the null hypothesis (H_0_) was supported when frequentist analyses were nonsignificant (Lakens et al., 2020). The prior of the Bayesian statistics in the analyses was set as default (null hypothesis), assuming the experimental variables had no effect on the dependent variables. The *BayesFactor* package was used to calculate Bayes factors (Morey et al., 2015).

### Results

We first report toddlers' proportion face looking (looking to face AOI/looking to all four AOIs looking) and target looking (looking to target AOI/looking to three object AOIs looking) in the referent selection phase to uncover how labeling affect influenced toddlers' attention distribution to novel objects during learning. Second, we analyzed toddlers' proportion target looking (looking to target AOI/looking to all three AOIs) and pointing data in the retention phases to test whether participants retained label‐object and emotion‐object associations differently based on the labeling affect associated with objects, and whether retention differed across the two retention phases.

#### Referent selection

Thirty‐two out of 38 participants' eye‐tracking data entered the analyses of the referent selection phase after data cleaning. Data from six participants who looked for less than 50% of every trial were removed. The dependent variables were toddlers' proportion face looking and proportion target looking following the first label onset until the trial end in the LMEMs reported below.

##### Proportion face looking

We first submitted proportion face looking and to a LMEM with a fixed effect of affect (neutral, positive, and negative). The best‐fitting random effect structure included by‐item, by‐participant, and by‐affect random intercepts, *χ* ^2^(1) = 28.65, *p* < .001. Toddlers' proportion face looking after label onset differed according to the labeling affects in referent selection trials, *χ* ^2^(2) = 59.11, *p* < .001, $R_{m}^{2}$ = .20, $R_{c}^{2}$ = .59. Their proportion face looking in the neutral referent selection trials (*M* = .44, *SD* = .18) was lower than that in the negative and positive referent selection trials (negative: *M* = .66, *SD* = .18; *β* = .21, *SE* = .03, *z* = 7.68, *p* < .001; positive: *M* = .56, *SD* = .16; *β* = .11, *SE* = .03, *z* = 4.05, *p* < .001). Post hoc Tukey's HSD tests indicated that the proportion face looking in the negative referent selection trials was greater than that of positive referent selection trials (*β* = .10, *SE* = .03, *z* = 3.52, *p* = .001).

##### Proportion target looking

We then submitted proportion target looking to a LMEM with a fixed effect of affect (neutral, positive, and negative). The best‐fitting random effect structure included by‐item and by‐participant random intercepts. Toddlers showed similar patterns of looking to targets labeled in the different affects (*χ* ^2^(2) = 3.44, *p* = .18, $R_{m}^{2}$ = .02, $R_{c}^{2}$ = .15, BF_01_ = 1.54, neutral: *M* = .78, *SD* = .21; positive: *M* = .82, *SD* = .15; negative: *M* = .85, *SD* = .18).

Overall, therefore, during the referent selection phase, toddlers looked to the neutral face the least and to the negative face the most; meanwhile, they attended to the target objects similarly between labeling affect.

#### Retention

**FIGURE 4:**
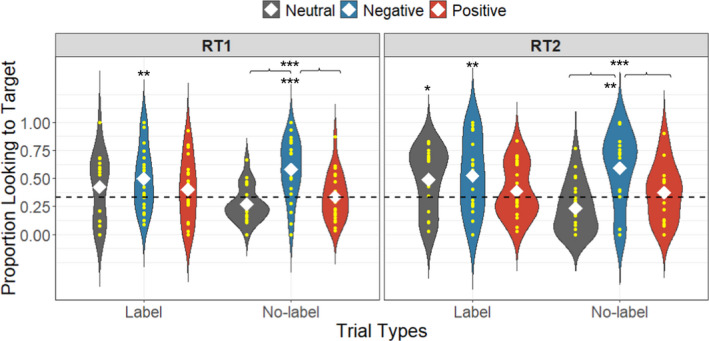
Proportion target looking in the retention phases in Experiment 1. White diamonds indicate the means of proportion target looking, and the dashed line represents chance (0.33). *p < .05, **p < .01, ***p < .001

At test, on label trials toddlers heard all targets labeled in neutral affect, and on no‐label trials toddlers heard the three emotional cue types. After data cleaning, data from 29 participants entered the looking time analyses of RT1 and data from 28 participants for RT2. To analyze toddlers' retention of label‐object and emotion‐object associations, we compared proportion target looking in a 6500 ms time window after the first label onset in both label and no‐label trials against chance (0.33) with a two‐tailed, one‐sample *t*‐test (Figure 4). The time window was chosen to ensure toddlers' fixations after hearing all the labels or cues were fully collected (Okumura et al., 2017; Twomey et al., 2017). Pointing from 30 participants entered the analyses in both retention phases. The probability of most pointing to one object, either target or distractors in each retention trial, was compared with random pointing (0.33) using the Chi‐Square Goodness‐of‐Fit Test (Howell, 2013). We coded points as *target* and *positive/negative/neutral distractor* (Table 1).

**TABLE 1:** Toddlers' target and distractor pointing in the retention phases

Affect	RT1				*χ* ^2^(2)	RT2				*χ* ^2^(2)
	Target	Distractor (*n*)				Target	Distractor (*n*)			
	*n*(*N*)	Neutral	Positive	Negative		*n* (*N*)	Neutral	Positive	Negative	
Label trials										
Neutral	23 (29)	—	5	1	28.41***	20 (28)	—	3	5	18.5***
Positive	12 (27)	10	—	5	2.89	11 (29)	10	—	8	0.48
Negative	20 (28)	6	2	—	19.14***	21 (28)	2	5	—	22.36***
No‐label trials										
Neutral	4 (22)	—	5	13	6.64*	4 (26)	—	5	17	12.08***
Positive	8 (22)	3	—	11	4.45	6 (27)	6	—	15	6.00*
Negative	21 (25)	3	1	—	29.12***	23 (28)	4	1	—	30.50***

*Note*: *N* is the total number of toddlers who pointed in a particular RT trial. *n* is the number of toddlers who pointed to a particular object. The percentage of toddlers' pointing is reported in the main text.

* *p* < .05

*** *p* < .001.

##### Label trials in RT1

Toddlers did not look at neutral and positive targets at levels greater than expected by chance (neutral: *M* = .42, *SD* = .30, *t*(15) = 1.25, *p* = .23, 95% CI [.26, .58], *d* = 0.30, BF_01_ = 2.04; positive: *M* = .40, *SD* = .28, *t*(18) = 1.07, *p* = .30, 95% CI [.26, .54], *d* = 0.23, BF_01_ = 2.56). However, they looked at negative targets at above‐chance levels (*M* = .50, *SD* = .28, *t*(22) = 2.91, *p* = .008, 95% CI [.38, .62], *d* = 0.59). A LMEM with a fixed effect of affect and random intercepts for items and participants indicated no differences in proportion target looking between the different emotional affects, *χ* ^2^(2) = 1.58, *p* = .45, $R_{m}^{2}$ = .02, $R_{c}^{2}$ = .13, BF_01_ = 348.59.

###### Pointing

In total, 79.31% and 71.43% of toddlers pointed to neutral and negative targets, respectively, above chance, but only 44.44% of toddlers pointed to positive targets.

##### Label trials in RT2

On the second day, toddlers looked to neutral and negative targets at levels greater than expected by chance (neutral: *M* = .49, *SD* = .28, *t*(18) = 2.54, *p* = .02, 95% CI [.36, .62], *d* = 0.57; negative: *M* = .52, *SD* = .33, *t*(20) = 2.67, *p* = .01, 95% CI [.37, .67], *d* = 0.57), whereas they did not look at positive targets at above‐chance levels (*M* = .39, *SD* = .24, *t*(19) = 1.06, *p* = .30, 95% CI [.28, .50], *d* = 0.22, BF_01_ = 2.63). A LMEM with a fixed effect of affect and random intercepts for items and participants indicated no differences in proportion target looking between the different emotional affects, *χ* ^2^(2) = 3.21, *p* = .20, $R_{m}^{2}$ = .05, $R_{c}^{2}$ = .18, BF_01_ = 384.41.

###### Pointing

In total, 71.43% and 75.00% of toddlers pointed to neutral and negative targets, respectively, above chance; but only 37.93% of toddlers pointed to positive targets.

Although toddlers did not look to the neutral target above chance in RT1, a significant amount of them pointed to the neutral target. We return to this discrepancy between looking and pointing in the Discussion. Overall, then, toddlers retained neutral and negative but not positive label‐object associations.

##### No‐label trials in RT1

After hearing neutral and positive cues, toddlers did not look at the corresponding targets at above‐chance levels (neutral: *M* = .27, *SD* = .16, *t*(21) = −1.76, *p* = .09, 95% CI [.20, .34], *d* = −0.40, BF_01_ = 1.19; positive: *M* = .34, *SD* = .20, *t*(22) = 0.16, *p* = .87, 95% CI [.25, .43], *d* = 0.02, BF_01_ = 4.52); but after hearing negative cues, they did look to negative targets at above chance levels (*M* = .58, *SD* = .26, *t*(25) = 4.90, *p* < .001, 95% CI [.47, .68], *d* = 0.95).

A LMEM with a fixed effect of affect and random intercepts for items and participants indicated that toddlers' proportion target looking differed between the different emotional affects, *χ* ^2^(2) = 29.25, *p* < .001, $R_{m}^{2}$ = .29, $R_{c}^{2}$ = .29. Planned post hoc Tukey's HSD tests indicated that toddlers looked to the negatively cued targets more than to both the neutrally and positively cued targets (neutral: *β* = .31, *SE* = .06, *z* = 5.07, *p* < .001; positive: *β* = .24, *SE* = .06, *z* = 4.02, *p* < .001); their looking to the positively and neutrally cued targets was not different (*β* = .07, *SE* = .06, *z* = 1.06, *p* = .54, BF_01_ = 1.88).

###### Pointing

Only 18.18% of toddlers pointed to neutral targets but 59.09% pointed to negative distractors, which is above chance; only 36.36% pointed to positive targets; and 84.00% of toddlers pointed to negative targets, which is above chance.

##### No‐label trials in RT2

On the second day, after hearing neutral and positive cues, toddlers did not look at the corresponding targets at above‐chance levels (neutral: *M* = .23, *SD* = .22, *t*(21) = −2.00, *p* = .06, 95% CI [.13, .33], *d* = 0.44, BF_01_ = 0.84; positive: *M* = .37, *SD* = .25, *t*(16) = 0.72, *p* = .48, 95% CI [.24, .50], *d* = 0.16, BF_01_ = 3.30); but again, after hearing the negative cue, they looked to the negative target above chance (*M* = .59, *SD* = .33, *t*(17) = 3.36, *p* = .004, 95% CI [.43, .75], *d* = 0.78).

A LMEM with fixed effect of affect and random intercepts for items, participants, and affect (*χ* ^2^(1) = 5.27, *p* = .02) indicated that toddlers' proportion target looking differed by affect, *χ* ^2^(2) = 18.34, *p* < .001, $R_{m}^{2}$ = .25, $R_{c}^{2}$ = .25. Planned post hoc Turkey's HSD test indicated that toddlers' looking to negatively cued targets was greater than to neutrally and positively cued targets (neutral: *β* = .36, *SE* = .08, *z* = 4.27, *p* < .001; positive: *β* = .22, *SE* = .09, *z* = 2.44, *p* = .04); their looking to the positively and neutrally cued targets was not different (*β* = .14, *SE* = .08, *z* = 1.65, *p* = .22, BF_01_ = 0.88).

###### Pointing

Only 15.38% toddlers pointed to neutral targets but 65.38% pointed to the negative distractor, which is above chance; only 22.22% pointed to positive targets but 55.56% pointed to the negative distractor, which is above chance; and 82.14% of toddlers pointed to negative targets, which is above chance.

##### Comparison of target looking across trial types between retention phases

A LMEM with the dependent variable of proportion target looking, fixed effects of an interaction of affect, retention phases (RT1 and RT2), and trial type (label and no‐label) and random intercepts for items, participants, and affect (*χ* ^2^(1) = 13.03, *p* < .001) revealed a significant effect of the interaction, *χ* ^2^(11) = 47.43, *p* < .001, $R_{m}^{2}$ = .16, $R_{c}^{2}$ = .17. However, planned post hoc Tukey's HSD tests indicated that proportion target looking time was not different across trial types and between the two retention phases based on affect, all *p*s > .06 (see Supporting Information).

Overall, toddlers looked and pointed to the negative target after hearing negative cues in both retention phases. After hearing neutral cues, they did not look to the neutral target but pointed to the negative distractor. However, they looked and pointed to the three objects randomly after hearing the positive cues despite more than half of them pointing to the negative distractor in RT2, indicating that toddlers tended to attend more to negative objects in the no‐label trials.

### Discussion

Experiment 1 examined the effect of labeling affect on toddlers' encoding and retention of novel label‐object associations and whether they had associated objects with the affect used in the referent selection phase. Our hypothesis was that toddlers would retain positively and negatively trained associations better than the neutral ones. We found that they showed robust evidence of retaining the negatively trained label‐object associations via looking and pointing on both days, and although they did not look to the neutral targets above chance on the first day, they looked and pointed to them on the second day. In contrast, toddlers failed to identify the positive target on both days after hearing the corresponding labels. In the no‐label trials, toddlers identified the negatively trained objects but not the neutrally or positively trained objects; notably, most of them pointed at the negative distractor after hearing neutral cues on both days, and after hearing positive cues on the second day. Together, these results show evidence for the retention of neutrally and negatively trained label‐object associations but not positive associations, while toddlers attended more to negative objects when hearing emotional cues. We interpret the effect of perceived emotions during learning on toddlers' retention of label‐object mappings in terms of a negativity bias (Hoehl, 2014; Vaish et al., 2008) and in addition, the effect of salient distractors on successful retention (Pomper & Saffran, 2018; Shafer & Dolcos, 2012).

Specifically, in line with studies that reported a negativity bias in emotional information processing in infants (e.g., see Bowen et al., 2018 for a review; Carver & Vaccaro, 2007; Moses et al., 2001), it is not surprising that toddlers looked and pointed at the negatively trained object after hearing the corresponding label and cue, which is also in accordance with findings in adults that negative emotional information is recognized better when it is targeted (Shafer & Dolcos, 2012). However, when the negative emotional information is a competitor, it can impair the ongoing attentional and cognitive processing (Eastwood et al., 2003). In the current case, the negativity bias during retention trials would direct attention to negative competitors of neutral and positive targets. Indeed, after hearing neutral and positive cues in the no‐label trials, toddlers did not look to the targets but pointed to the negative distractors. However, this situation happened only when toddlers heard neutral and positive cues, and not labels.

To understand different looking and pointing results in the label trials, we need to know how toddlers can demonstrate successful retention of label‐object associations: After hearing the object labels, toddlers need to integrate linguistic (the label) and perceptual (the referent) cues and then adjust and sustain attention to the target among a set of distractors (Samuelson & Smith, 2000). This process can be promoted when toddlers are familiar with the referent (Kucker & Samuelson, 2012) but interrupted by the presence of salient distractors (e.g., familiar object salience: Pomper & Saffran, 2018). In the current case, the negative object could be the most salient among the three objects. Additionally, the cognitive load of retrieving positively and negatively trained associations is higher because in the label retention trials toddlers heard all labels presented in neutral affect. As the result, they were required to generalize novel labels across emotional affect (Singh et al., 2004). In contrast, on neutral label trials, no generalization was necessary, which lowered the task demands for neutrally trained label‐object associations. Therefore, despite toddlers failing to sustain their looking to above‐chance levels for neutral label‐object associations, the majority of them still pointed to the neutral targets. In positive retention trials, by contrast, toddlers were faced with two difficulties: not only was there a negative distractor, but now they also had to generalize the label to neutral affect.

We take two insights from these considerations: first, target looking might suffer interference in the presence of a negative competitor. Second, target looking and, to an extent, pointing are a function of target and distractor salience: For the label trials, target salience was reduced in positive trials because affect at training and test differed, which was not the case for neutral targets; for the no‐label trials, the salience of the negative distractor may have overpowered the effect of neutral or positive cues on attention.

To test the assumption that distractor salience masked retention by reducing attention to the target item, we conducted a second experiment in which the retention trials contained only one distractor, so that positive targets occurred in trials without neutral and negative distractors, respectively. Furthermore, in no‐label retention trials, we only used neutral affect in order to better understand the effect of high‐affect objects (positive and negative) on visual attention.

## EXPERIMENT 2

Experiment 2 aimed to replicate the retention of label‐object associations in Experiment 1 for neutral and negative targets while examining whether, in Experiment 1, toddlers' failure to look and point above chance at positive targets indicated a true lack of retention of this mapping or was due to the influence of the distractor. We hypothesized that toddlers would show above‐chance target looking for all targets, indicating retention of label‐object mappings irrespective of affect.

### Method

#### Participants

Forty 29‐ to 31‐month‐old typically developing, white British monolingual English‐learning toddlers (16 girls, *M* = 30.50 months, *SD* = 0.72 months) participated in the study. An additional 13 toddlers were excluded from analyses for fussiness, as defined by failure to remain on the caregiver's lap, (7); caregiver intervention (3); eye tracker error (2); and experimenter error (1). Toddlers' mean productive vocabulary was 353.27 (*SD* = 57.89, range = 164–412, the 50th, 75th, and 90th percentiles are 377, 396, and 402, respectively), above the 75th percentile of the norm (Hamilton et al., 2000). Caregivers gave consent to participate and received travel reimbursement. Toddlers were given a story book for taking part.

#### Procedure and design

The procedure of Experiment 2 was identical to Experiment 1: a referent selection phase followed by a 5‐min break, then RT1, with participants completing RT2 on the following day. However, we adapted the design: first, referent selection trials in which the novel objects were targets were 20 s long, with the four label onsets occurring at 8000, 10,300, 13,000, and 18,000 ms after trial onset. Second, presentation order of neutral, positive, and negative blocks within the referent selection phase was counterbalanced.

**FIGURE 5:**
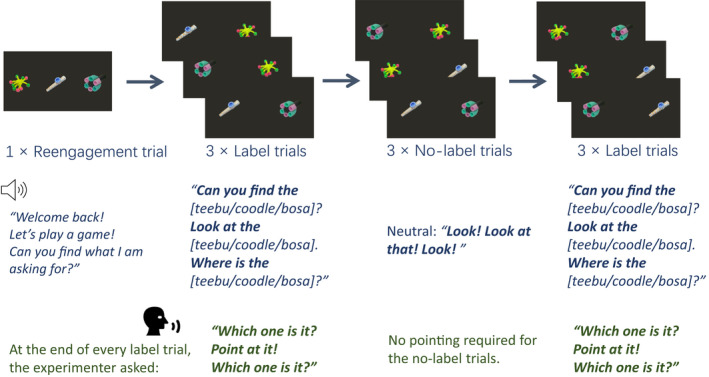
An example of retention trials in Experiment 2. For a video example of the retention phase (see https://doi.org/10.17605/OSF.IO/72KPS)

In the retention phases (Figure 5), in contrast to Experiment 1, only two out of the three novel objects were presented in each retention trial to delineate the impact of labeling affect and distractor items on looking times and pointing. Each retention phase consisted of three blocks: two blocks of three label trials were presented in the first and the third blocks, while one block of three no‐label trials was presented in the second block. Three object pairs were presented in each block: *neutral–positive*, *neutral–negative*, and *negative–positive*. In line with the label trials in Experiment 1, the target was labeled three times in neutral affect on each label trial (e.g., *Can you find the coodle*? *Look at the coodle*. *Where is the coodle*?). Each object served as the target once in each block. Additionally, participants were also encouraged to point at the target object after the last label onset. Different from the no‐label trials in Experiment 1, Experiment 2 presented three no‐label trials in which cues were presented in a neutral tone (*Look! Look at that! Look!*). Participants were not asked to point because no target was specified.

Thus, in Experiment 2, each retention phase consisted of a total of six label trials and three no‐label trials. Object pairings and left–right combinations were Latin square counterbalanced. Trial order was pseudorandomized within blocks to ensure the target object was presented on the same side in no more than two consecutive trials and the objects in the first no‐label trial were not targets in the previous label trial. Label and neutral cue onsets on each retention trial were at 2000, 4000, and 6000 ms from the beginning of a trial; trial length was 8000 ms.

#### Data cleaning and model selection

As in Experiment 1, trials in which participants' looking was less than 50% of the trial duration were excluded from analyses. In total, 311 out of 333 trials were included for the analyses of the referent selection phase (93.39%); 235 out of 333 trials for RT1 (70.57%) and 239 out of 333 trials for RT2 (71.77%). Toddlers' pointing in the label trials were recorded by the experimenter. The pointing was coded as the first point location (left and right) after the third label onset. The pointing from 37 toddlers who pointed on more than two trials in each retention phase entered analyses (398 trials). A second coder, naïve to the experimental hypotheses, additionally coded 50% of the recordings. Intercoder reliability was high (Cohen's *κ*: .89). The approach to model selection was in line with Experiment 1.

### Results

#### Referent selection

Thirty seven out of 40 participants' eye‐tracking data entered into the analyses of the referent selection phase after data cleaning. Data from three participants were removed because of looking less than 50% on every trial.

##### Proportion face looking

We first submitted proportion face looking to a LMEM with a fixed effect of affect. The best‐fitting random effect structure included by‐item, by‐affect random intercepts, and by‐participant random slopes for affect, *χ* ^2^(5) = 29.58, *p* < .001. Toddlers' proportion face looking after label onset differed according to the labeling affects in referent selection trials, *χ* ^2^(2) = 18.34, *p* < .001, $R_{m}^{2}$ = .08, $R_{c}^{2}$ = .57. Compared with the proportion face looking in the neutral referent selection trials (*M* = .55, *SD* = .16), that in the negative referent selection trials was higher (*M* = .65, *SD* = .19; *β* = .14, *SE* = .03, *z* = 3.57, *p* = .001), but similar in the positive referent selection trials (*M* = .55, *SD* = .15; *β* = .004, *SE* = .02, *z* = 0.19, *p* = .98). Post hoc Tukey's HSD tests indicated that the proportion face looking in the negative referent selection trials was higher than that of positive referent selection trials (*β* = .10, *SE* = .02, *z* = 4.21, *p* < .001).

##### Proportion target looking

We then submitted proportion target looking to a LMEM with a fixed effect of affect and by‐item, by‐participant random intercepts, and by‐participant random slopes for affect (*χ* ^2^(5) = 12.93, *p* = .02). Toddlers' proportion target looking was similar across labeling affect (*χ* ^2^(2) = 4.24, *p* = .12, $R_{m}^{2}$ = .03, $R_{c}^{2}$ = .37, BF_01_ = 0.46; neutral: *M* = .76, *SD* = .17; positive: *M* = .83, *SD* = .19; negative: *M* = .80, *SD* = .17). Similar to Experiment 1, toddlers looked to the neutral face the least, and the negative face the most, and did not show different target looking among the three target objects across labeling affect.

#### Retention

**FIGURE 6:**
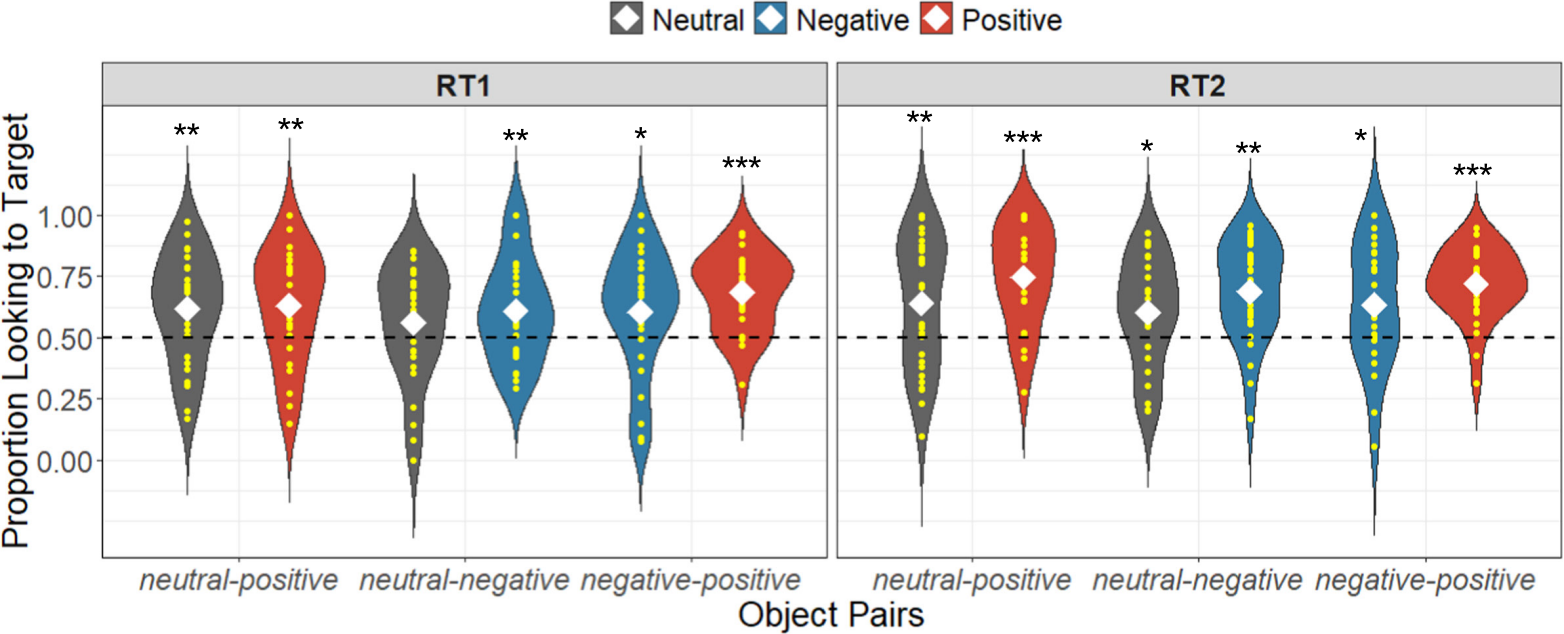
Proportion target looking in label trials in the retention phases in Experiment 2. White diamonds indicate the means of proportion target looking, and the dashed line represents chance (0.50). *p < .05, **p < .01, ***p < .001

**FIGURE 7:**
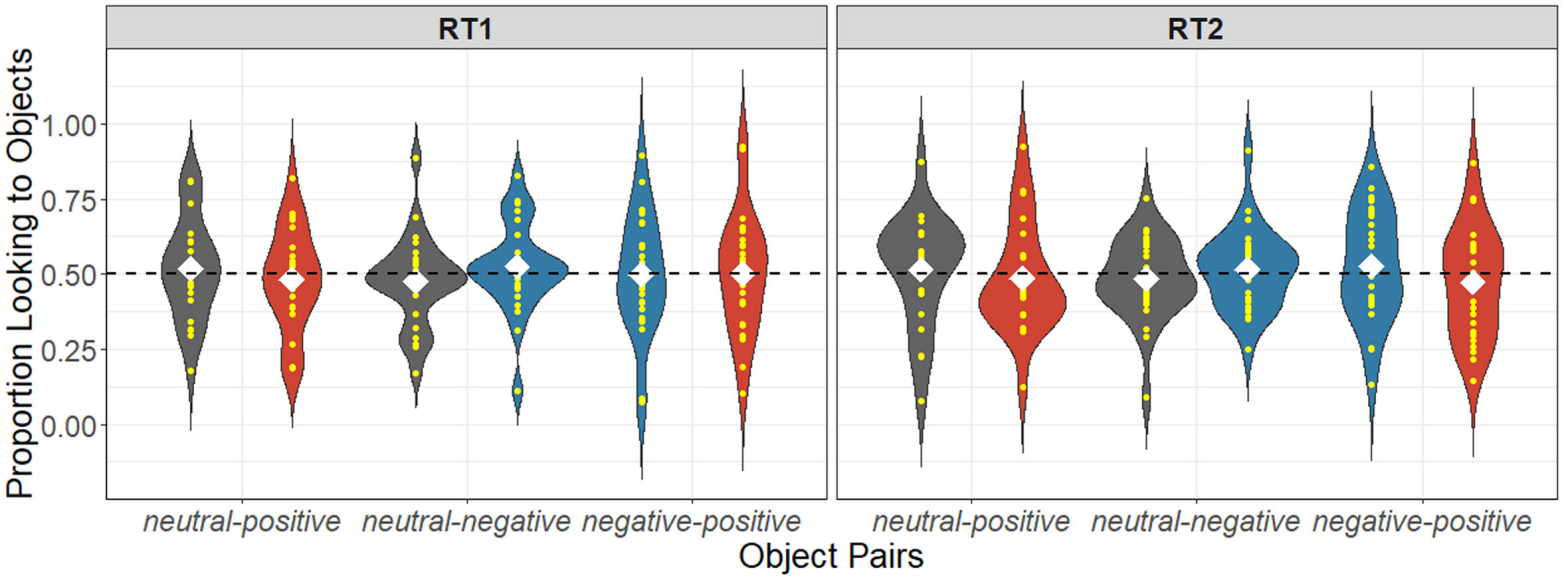
Proportion object looking in no‐label trials in the retention phases. Toddlers only heard *neutral* cues, *look*. White diamonds indicate the means of proportion target looking, and the dashed line represents chance (0.50). *p < .05, **p < .01, ***p < .001

After data cleaning, data from 37 participants entered the analyses for RT1 and 36 entered the analyses for RT2. Proportion looking to a given object was again calculated across the 6500 ms time window after the first label onset. For label trials, proportion looking to the target was compared against chance (0.50) using one‐sample *t*‐tests (two‐tailed; Figure 6), as was proportion looking to each object in each object pair on no‐label trials (Figure 7). Pointing from 36 participants entered the analyses for RT1 and 37 entered the analyses for RT2. Points to the target or distractor on each label trial were compared with random pointing (0.50) by binominal test. We coded points as *target* and *positive/negative/neutral distractor* (Table 2).

**TABLE 2:** Binominal test of toddlers' target pointing in label trials

Object pair	RT1			RT2		
	Target *n* (*N*)			Target *n* (*N*)		
	Neutral	Positive	Negative	Neutral	Positive	Negative
Neutral‐positive	23 (29)**	27 (33)***	—	29 (36)***	29 (33)***	—
Neutral‐negative	27 (34)***	—	24 (32)**	30 (35)***	—	29 (36)***
Negative–positive	—	27 (29)***	26 (31)***	—	29 (36)***	27 (34)***

*Note*: *N* is the total number of toddlers who pointed in a trial. *n* is the number of toddlers who pointed to a specific object. The percentage of toddlers' pointing is reported in the main text.

** *p* < .01

*** *p* < .001.

##### Label trials in RT1

Aside from the neutral target in the *neutral‐negative* pairs, toddlers looked to the targets at above‐chance level.

###### Neutral‐positive pairs

Neutral objects as target: *M* = .62, *SD* = .22, *t*(27) = 2.88, *p* = .008, 95% CI [.53, .71], *d* = 0.54; 79.31% of toddlers pointed to neutral targets, above chance. Positive object as target: *M* = .63, *SD* = .23, *t*(23) = 2.96, *p* = .01, 95% CI [.53, .73], *d* = 0.56); 81.82% of toddlers pointed to positive targets, at above‐chance levels.

###### Neutral‐negative pairs

Neutral objects as target: *M* = .56, *SD* = .23, *t*(29) = 1.43, *p* = .16, *d* = 0.26, 95% CI [.47, .65], BF_01_ = 2.06), but 79.41% of toddlers pointed to neutral targets, above chance. Negative objects as target, *M* = .61, *SD* = .21, *t*(29) = 2.89, *p* = .007, 95% CI [.53, .69], *d* = 0.53); 75.00% of them also pointed to the targets, above chance.

###### Negative–positive pairs

Positive objects as target: *M* = .68, *SD* = .16, *t*(23) = 5.60, *p* < .001, 95% CI [.62, .75], *d* = 1.14; 93.10% of toddlers pointed to positive targets, above chance. Negative objects as target: *M* = .60, *SD* = .25, *t*(25) = 2.11, *p* = .045, 95% CI [.50, .70], *d* = 0.41); 83.87% of toddlers pointed to negative targets, above chance.

A LMEM with an interaction of affect (neutral, positive, and negative) and object pairs (*neutral‐positive*, *neutral‐negative*, and *negative–positive*) and random intercepts for items and participants indicated no differences in proportion target looking between different affects across the object pairs, *χ* ^2^(5) = 4.66, *p* = .46, $R_{m}^{2}$ = .03, $R_{c}^{2}$ = .12, BF_01_ = 18,184.62.

##### Label trials in RT2

Toddlers looked above chance to all targets in all the object pairs.

###### Neutral‐positive pairs

Neutral object as target: *M* = .64, *SD* = .27, *t*(29) = 2.91, *p* = .007, 95% CI [.54, .74], *d* = 0.53; 80.56% of toddlers pointed to the neutral target, above chance. Positive object as target: *M* = .75, *SD* = .21, *t*(21) = 5.49, *p* < .001, 95% CI [.65, .84], *d* = 1.17; 87.88% of toddlers pointed to positive targets, above chance.

###### Neutral‐negative pairs

Neutral object as target: *M* = .60, *SD* = .22, *t*(24) = 2.34, *p* = .03, 95% CI [.51, .96], *d* = 0.47; 85.71% of toddlers pointed to neutral target, above chance. Negative object as target: *M* = .69, *SD* = .20, *t*(28) = 5.06, *p* < .001, 95% CI [.61, .76], *d* = 0.94; 80.56% of toddlers pointed to negative targets, above chance.

###### Negative–positive pairs

Positive object as target: *M* = .72, *SD* = .14, *t*(29) = 8.29, *p* < .001, 95% CI [.66, .77], *d* = 1.51; 80.56% of toddlers pointed to positive target, above chance. Negative object as target: *M* = .64, *SD* = .25, *t*(23) = 2.62, *p* = .02, 95% CI [.53, .74], *d* = 0.53; 79.41% of toddlers pointed to negative targets, above chance.

A LMEM with an interaction of affect and object pairs and random intercepts for items and participants indicated no differences in proportion target looking between the different affects across the object pairs, *χ* ^2^(5) = 9.91, *p* = .08, $R_{m}^{2}$ = .05, $R_{c}^{2}$ = .21, BF_01_ = 352.54.

##### Comparison of target looking between retention phases

A further LMEM with an interaction of affect, object pairs, and retention phase and random intercepts for items, participants, and affect (*χ* ^2^(1) = 4.27, *p* = .04) revealed a significant result of the interaction, *χ* ^2^(11) = 21.96, *p* = .02, $R_{m}^{2}$ = .05, $R_{c}^{2}$ = .20. However, a planned post hoc Tukey's HSD test indicated that the proportion target looking time was not different across object pairs between the two retention phases based on affect, all *p*s > .76 (see Supporting Information).

Overall, aside from toddlers not looking to the neutral target when it was paired with a negative distractor in RT1, toddlers looked and pointed to targets in all the label‐object associations in both retention phases.

##### No‐label trials

Overall, after hearing neutral cues, toddlers did not look systematically to any of the objects in each object pair in both retention phases. A further time‐bin analyses is reported in Supporting Information to explore toddlers' detailed looking patterns.

###### RT1

For *neutral‐positive* pairs (neutral: *M* = .52, *SD* = .17; positive: *M* = .48, *SD* = .17, *t*(22) = −0.51, *p* = .61, 95% CI [.41, .56], *d* = 0.11, BF_01_ = 4.05). For *neutral‐negative* pairs (neutral: *M* = .47, *SD* = .15; negative: *M* = .52, *SD* = .15, *t*(24) = 0.81, *p* = .42, 95% CI [.46, .59], *d* = 0.16, BF_01_ = 3.51). For *negative–positive* pairs (positive: *M* = .50, *SD* = .20; negative: *M* = .50, *SD* = .20, *t*(22) = −0.04, *p* = .97, 95% CI [.41, .59], *d* = 0.007, BF_01_ = 4.57).

###### RT2

For *neutral‐positive* pairs (neutral: *M* = .51, *SD* = .19; positive: *M* = .49, *SD* = .19, *t*(20) = −0.34, *p* = .74, 95% CI [.40, .57], *d* = 0.07, BF_01_ = 4.17). For *neutral‐negative* pairs (neutral: *M* = .48, *SD* = .13; negative: *M* = .51, *SD* = .13, *t*(26) = 0.60, *p* = .56, 95% CI [.46, .57], *d* = 0.11, BF_01_ = 4.17). For *negative–positive* pairs (positive: *M* = .47, *SD* = .18; negative: *M* = .53, *SD* = .18, *t*(26) = 0.78, *p* = .44, 95% CI [.46, .60], *d* = 0.15, BF_01_ = 3.73).

### Discussion

Experiment 2 was designed, first, to clarify whether toddlers' failure to identify positive label‐object associations in Experiment 1 was because of a lack of retention or the influence of salient distractors. The results supported the latter interpretation: With only one distractor present, toddlers looked at the positive target above chance in all trials. Interestingly, toddlers looked at above‐chance levels to the neutral target in the *neutral‐positive* pair but not in the *neutral‐negative* pair on the first day; however, they pointed at the neutral target in that pair. This result confirms what we found in Experiment 1: in addition to looking times, pointing provides an independent, potentially more robust measure of retention of newly learned label‐object associations when salient distractors are present. On the second day, toddlers looked and pointed at the neutral target when paired with a negative distractor, suggesting that the negativity bias in visual attention masking target looking was temporary. Overall, labeling affect did not influence word learning outcomes, with retention shown across all affects, although looking patterns were still affected by the salient negative distractor—an effect that diminished on day 2.

Second, we also explored whether toddlers' visual attention to objects was influenced by positive and negative affect associated with the objects. Different from 12‐month‐old infants preferring positively to negatively habituated objects (Flom & Johnson, 2011), 30‐month‐old toddlers showed no visual preference for any objects in the current study. In sum, Experiment 2 suggests that toddlers' failure to identify the positive label‐object association in Experiment 1 can be explained by the effect of distractors on toddlers' visual attention, which was diminished when only one distractor was present.

## GENERAL DISCUSSION

The current research studied how toddlers integrate linguistic and affective cues during word learning by investigating whether labeling objects with different emotional affect influenced 30‐month‐olds' learning and retention of these label‐object associations and whether toddlers formed associations between objects and perceived emotions. Compatible with theories arguing that children perceive and capitalize on multiple information sources available in their environment when learning label‐object associations (e.g., Hollich et al., 2000; Monaghan et al., 2018; Yu & Ballard, 2007), we further found that toddlers' attentional processing was affected by linguistic and emotional cues. In particular, they were influenced by linguistic cues over emotional cues when learning words: While their attention was attracted to negative affect, children nonetheless learned novel label‐object associations. We discuss these findings in terms of the impact of perceived emotions on referent selection, retention, and the possible impact of experimental factors, timing of testing, on performance of memory retrieval of newly learned information.

### The impact of perceived emotions on referent selection

In the real world, early word learners acquire label‐object associations in social interactions, which are rich in emotional information that affects infants' perception and attention (Clark, 2016; Tomasello, 2003). In this study, we embedded the learning phase in neutral, positive, and negative emotional affect to mimic the situations that young word learners might encounter in their life. During the referent selection learning phase, negative facial expressions attracted more attention from 30‐month‐old toddlers than neutral and positive expressions, providing evidence that others' negative facial expressions elicit more visual attention while toddlers are encoding both faces and objects. Although toddlers spent similar amount of time processing the novel objects during learning visually, the negative object received above chance visual attention and pointing during retrieval based on both linguistic cues (labels) and affective cues (*urgh*), indicating that toddlers actively encoded others' affective expressions and linked them with the linguistic cues and objects they processed during referent selection.

### The impact of perceived emotions on retention

In the retention phases, to demonstrate successful retention of label‐object and emotion‐object associations, first, for the positively and negatively trained associations, toddlers must generalize from the positive/negative affect‐object associations encountered during referent selection, maintaining this association at test when the target object was accompanied by neutral affect; then, after hearing labels or affective cues, toddlers need to detect the corresponding targets and sustain their looking to them at above‐chance level or point to them. During this process, toddlers' identification of targets was affected by the linguistic and affective cues they heard, the newly learned emotionality of objects and, probably, timing of testing.

#### Linguistic and affective cues

As reported, toddlers' looking to targets at test was not different between when asking by labeling and when asking by affective cues, suggesting both types of cues direct a similar amount of toddlers' visual attention to the targets. However, toddlers were more likely to sustain their looking to targets at above‐chance level after hearing labels and negative cues than the neutral and positive cues. Regarding labels, infants as young as 12 months privilege words over other sounds in associative learning of objects (Althaus & Westermann, 2016; MacKenzie et al., 2011). In particular, children understand the grammatical structure of linguistic cues and map *bosa* to the novel object after hearing *this is a bosa!* in referent selection (Hollich et al., 2000). Similarly, 13‐ and 18‐month‐olds associated both words and nonlinguistic sounds with objects when they were instructed *Look at what you have! [word/sound] That is what we call that one!* (Campbell & Namy, 2003). In contrast, the affective cues used in this study (*look*, *wow*, *urgh*) were attributed to the novel objects via the actress' expressions in referent selection (*Wow! Look! This is bosa!*) instead of calling the objects *wow* or *urgh* directly. Thus, labels serve as better referential cues relative to affective cues in identifying an object as the target.

Regarding affective cues, the negativity bias found in Experiment 1 suggests that the negatively trained object grabbed toddlers' attention. There are two possible accounts for this increased salience of the negative object: first, the evolutionary meaning of disgust, such as detecting a potential toxic object, makes toddlers process the negative object deliberately (Curtis et al., 2011). Second, less experience of others' negative expressions in toddlers' daily life than neutral or positive expressions makes the negative object more novel (Lieberman, 2017). Thus, toddlers link negative cues with the object more robustly than the neutral and positive cues, and the negative objects captured more attention.

#### Object emotionality

While a cue may explicitly drive toddlers' recall of a target associated with the cue, the distractors paired with the target can nonetheless have an implicit effect on toddler's attentional distribution. Specifically, on label trials, failure to identify positive targets in Experiment 1 was due to high cognitive load: the requirement of generalizing the spoken label from positive to neutral tone and suppressing the effect of a negativity bias on attention. In contrast, cognitive load was lower at test in Experiment 2 as one distractor was removed, reducing the implicit impact of the negativity bias. But this change also influenced toddlers' looking patterns in the no‐label trials. Statistically speaking, in the presence of a single distractor, toddlers must sustain longer looking to demonstrate above chance looking times, which could account for the overall chance looking in the no‐label trials of Experiment 2. In other words, the two‐alternative force choice task may not be sensitive enough to measure the impact of object emotionality on visual attention.

#### Timing of testing

Regarding the timing of testing, both newly learned words and emotional information are sleep consolidated (e.g., Lewis et al., 2011; Williams & Horst, 2014), thus the links between labels, perceived emotions, and objects should be strengthened a day after learning. First, in neutral label trials with a negative distractor presented in Experiment 1, toddlers' sustained visual attention increased to above‐chance level on the second day, suggesting that the effect of labeling dominates the effect of negative object emotionality in long‐term retrieval. Second, the above chance pointing to negative objects in all the no‐label trials of Experiment 1 on the second day further confirms the salience of the negative object when no linguistic cues presented. Combining these two points, we suggest a competitive process during retention in which the negatively trained object competes for attention, but hearing the labels facilitates the identification of corresponding targets.

### Limitations and future directions

First, the neutral cue (*Look*) in the current studies is generally used to ask people to look at a particular thing, thus it may not be the best representation of neutral affect to examine whether toddlers associate neutral affect with the object; a usage of “*Umm*” might be more suitable to build neutral affect‐object association, that is, *Umm*, *this is bosa* instead of *Look! This is bosa*. Second, toddlers' pointing was collected to reflect the outcome of the selection process when identifying targets. But the lack of pointing in no‐label trials of Experiment 2 led to an unexplored question in this study: whether toddlers' attention bias varies based on the emotionality of objects, or a negativity bias remains in a forced choice task. Moreover, there were discrepancies between toddlers' target looking and pointing, for example, the majority of toddlers in Experiment 1 pointed to negative objects after hearing all the affective cues, but their looking was above chance only for the negative object when hearing negative cues. Thus, an exploration on the relation between looking and pointing in early life could address possible different cognitive processes between looking and pointing (e.g., looking as implicit processing for information gathering, pointing as an explicit response).

Additionally, given that only neutral affect was employed in the no‐label trials in Experiment 2, the design could not reveal the possible effect that emotional affect might have on toddlers' object processing; including positive and negative affect in these trials would address this issue. Third, although the current findings suggest that toddlers prioritized labels over affective cues when identifying the referents, the measurement of eye tracking cannot further evaluate how linguistic and affective cues interact during information encoding, maintenance and retrieval in early development. This question could best be explored using brain imaging, such as comparing ERP responses of recognizing objects with emotional intonation with and without knowing their names.

Overall, the current research provides initial evidence on how perceived emotions affect the learning of label‐object associations when encountering multiple cues. When toddlers integrated linguistic and affective cues and objects during word learning, their acquisition of the novel label‐object associations was not influenced by the emotional expressions that they encountered during referent selection; rather, the procedure of retrieval of the associations was affected by the emotions associated with the objects. We conclude that referent selection during word learning is shaped by an interaction between the influence of labels, perceived emotions, and referents: labels, as linguistic cues, dominate the formation of label‐object associations; perceived emotions, as social pragmatic cues, shape the salience of objects. As a result, 30‐month‐old toddlers acquire label‐object associations irrespective of which emotions they perceive during learning. However, their attention is affected by others' negative emotion and the objects associated with it. Additionally, methodologically speaking, in situations with salient distractor items, participants' pointing can provide a more robust test of their knowledge than looking preferences.

## CONFLICT OF INTEREST

The authors have no conflict of interest to disclose.

## Supporting information


Data S1.
