# Supplementary material for: The impact of perceived emotions on toddlers' word learning
Source: Child Dev. 2022 May 30;93(5):1584–600. doi: 10.1111/cdev.13799 (PMC10108568; doi:10.1111/cdev.13799)
Supplement: Supplementary file 1 — Data S1. [file CDEV-93-1584-s001.docx]

Supplementary Materials

Experiment 1

Table S1 presents the size of all the object pictures, and the time course of video stimuli in the warm up and referent selection phases in Experiment 1.

| Table S1 | |  |  |  |  |  |
| --- | --- | --- | --- | --- | --- | --- |
| *Size of Object Pictures and Time Course of Video Stimuli in Warm up and Referent Selection Phases* | | | | | | |
| Phases Type | Target Stimuli (pixel) | Affect Types | Trial Length(ms) | 1st label onset | 2nd label onset | 3rd label onset |
| Warm up | apple (202 × 202) | Neutral | 15000 | 6800 | 8400 | 10200 |
|  | ball  (192 × 189) |  | 15500 | 6600 | 8200 | 10000 |
|  | banana  (242 × 196) |  | 16000 | 7000 | 8600 | 10400 |
|  | car  (245 × 198) |  | 15000 | 6900 | 8500 | 10200 |
|  | cup  (167 × 207) |  | 16000 | 7200 | 8800 | 10600 |
|  | flower  (211 × 208) |  | 15500 | 6700 | 8400 | 10200 |
| Referent Selection | bosa | Neutral | 15500 | 6700 | 8400 | 10100 |
|  |  | Positive | 17500 | 7350 | 9300 | 11400 |
|  | (241 × 201) | Negative | 20000 | 8150 | 10400 | 12750 |
|  | coodle | Neutral | 16000 | 6900 | 8400 | 10150 |
|  |  | Positive | 18500 | 7000 | 9100 | 11200 |
|  | (191 × 149) | Negative | 20000 | 7800 | 10100 | 13100 |
|  | teebu | Neutral | 15500 | 6700 | 8300 | 10100 |
|  |  | Positive | 19000 | 7400 | 9550 | 11750 |
|  | (268 × 211) | Negative | 21000 | 8050 | 10500 | 13200 |
| *Note.* Label onsets were counted from the trial onset. | | | | | | |

Detail Description of Video Stimuli

**Engagement stimuli.** In a six-second engagement video, the actress, shown at the top center of the screen, smiled and said *Hello! Let’s play a game! Can you find what I am asking for?* in child-directed speech in positive affect.

**Warm-up stimuli.** Two warm-up trials were designed to familiarize toddlers with the experimental procedure. The same actress appeared at the top center of the screen and three known objects appeared at the bottom left, central and right positions (e.g., *banana -cup - flower*). The positions of known objects were counterbalanced across participants. At the beginning of the trial, the actress turned her head to look at the three objects one by one with neutral affect, in silence. Then she looked forward and labeled one of the objects (e.g., *Can you find* *the* [*banana*]*? Look at the* [*banana*]! *Where is the* [*banana*]*?*). Afterwards, toddlers received ostensive feedback: the actress looked at and labeled the target object in a neutral, friendly manner: *Look! There is the* [*banana*], during which the target object (e.g., *banana*) increased in size.

**Referent selection phase.** The referent selection phase consisted of three blocks in which novel targets were labeled in either neutral, positive or negative affect, and all familiar targets were named in neutral affect. Each block consisted of five trials in which the same novel object and two familiar objects were presented. Sets for each block were *coodle-flower-ball*, *teebu-car-banana* and *bosa-apple-cup* (see Figure 2). Each familiar object served as the target object once (known trials) and each novel object served as the target three times per block (novel trials). Ostensive feedback was only given when novel objects were targets in the referent selection phase to facilitate retention (Axelsson, Churchley & Horst, 2012).

Block order and the horizontal position of objects in each trial were counterbalanced across participants. Trial order was pseudorandomized within each block with the constraint that the same trial types (known, novel) did not occur more than twice in succession. The first block was assigned neutral affect as a baseline for all participants. The order of positive and negative affect was counterbalanced across toddlers in the second and third blocks. Because the procedure and manner of the actress in neutral trials was identical to that in the warm-up trials, only positive and negative trials are described in detail.

***Positive trials.*** On positive trials, the actress first looked at the three objects one by one with an emotionally positive (happy) expression: she leaned her body forward with mouth open, eyebrows raised and looked at all objects happily in silence. Then, she looked ahead and labeled the novel objects in a cheerful voice (e.g., *Can you find* *the* [*bosa*]*? Look at the* [*bosa*]! *Where is the* [*bosa*]*?*). At the end of the trial, with a happy facial expression, she leaned her body towards the target object with a happy interjection (e.g., *Wow! Look! There is* *the* [*bosa*]!), during which the target increased in size.

***Negative trials.*** On negative trials, the actress first looked at the three objects one by one with an emotionally negative (disgusted) expression: she leaned backwards, frowned, wrinkled her nose and lifted her upper lip as she viewed the three objects in silence. Then, she looked ahead and labeled the objects in a nasal, tense, lower voice (e.g., *Can you find* *the* [*teebu*]*? Look at the* [*teebu*]! *Where is the* [*teebu*]*?*). At the end of the trial, with a disgusted facial expression, she leaned her body backwards away from the target object with a disgusted interjection (e.g., *Urgh! Look! There is the* [*teebu*]!), during which the target increased in size.

**Reengagement stimuli*.*** After the 5-minute break, in a seven-second reengagement video, the three novel objects were shown without the presence of the actress. Toddlers heard an audio recording of the same female actress saying *Welcome back!* *Let’s play a game! Can you find what I am asking for?* to help toddlers to reengage with the study.

**Retention phases.** In both retention phases (RT1, RT2), videos of six test trials consisted of three novel objects presented without the actress. The six test trials were divided into two blocks. One consisted of three label trials, and the other consisted of three no-label trials. The horizontal position of novel objects and trial order were counterbalanced across toddlers, and block order was reversed and counterbalanced in RT1 and RT2 across participants (see Figure 3). RT1 took place following the referent selection trials after a five-minute break. RT2 took place the following day within 36 hours of the toddlers completing referent selection and RT1.

***Label trials***. Label trials tested whether toddlers had retained the label-object associations. At the beginning of every trial, the three previously seen novel objects appeared accompanied by a chiming sound to attract toddlers’ attention. Then, toddlers heard audio recordings of the same actress labeling the objects in neutral affect (*Can you find the* [*label*]? *Look at the* [*label*]? *Where is the* [*label*]?).

***No-label trials***. No-label trials tested whether toddlers had associated the objects with the emotions displayed during referent selection. At the beginning of every trial, three novel objects appeared, again accompanied by a chiming sound. Then, toddlers heard emotional cues but not labels (Neutral: *Look! Look at that! Look!* Positive: *Wow*! *Look*! *Wow*! *Look at that*! *Wow*! Negative: *Urgh*! *Look*! *Urgh*! *Look at that*! *Urgh*!).

Participants’ Looking Patterns in Referent Selection

As the length of novel RS trials varied due to temporal characteristics of speech in the different emotions (Koolagudi et al., 2010) (Table S1), we employed linear mixed effects models (LMEMs) to examine whether the different trial lengths influenced participants’ raw looking time across entire trials and to targets in terms of labeling affects (neutral, positive and negative).

Analyses of Participants’ Raw Looking Time in referent selection trials

First, we submitted participants’ raw looking times over the entire trials to a LMEM with a fixed effect of affect and random intercepts for experimental items, participant and affect (*χ^2^*(1) = 57.92, *p* < .001). Toddlers’ raw looking time for a whole trial differed significantly by labeling affect, *χ^2^*(2) = 168.65, *p* < .001, *R^2^_m_ =* .45*, R^2^_c_ = .*60. Specifically, compared with toddlers’ looking time in neutral RS trials (*M* = 13373 ms, *SD* = 2341 ms), toddlers looked for longer in the positive and negative trials (positive: *M* = 15293 ms, *SD* = 2264 ms, *β* = 1917.20, *SE* = 395.70, *z* = 4.85, *p* < .001; negative: *M* = 18394 ms, *SD* = 2328 ms, *β* = 5012.30, *SE* = 398.10, *z* = 12.89, *p* < .001); toddlers also looked for longer in negative RS trials than in positive RS trials (*β* =3095.10, *SE* = 397.10, *z* = 7.80, *p* < .001). Thus, toddlers’ raw trial looking time was in line with the trial lengths.

Second, we submitted participants’ raw looking time to novel targets to a LMEM with a fixed effect of affect and random intercepts of by experimental items and by participants. Toddlers’ raw looking times to novel targets were not different between labeling affects, *χ^2^*(2) = 0.20, *p* = .91, *R^2^_m_ =* .0009*, R^2^_c_ = .*37, BF_01_ = 20.39. Overall, although toddlers’ raw looking time for entire trials differed according to labeling affects, their raw looking times to novel target objects remained similar.

Participants’ Looking Behaviors in Retention Phases

To further explore toddlers’ looking patterns in the retention phases, we first calculated their proportion target looking time of every 100ms time bin alongside the time course of label and no-label trials to illustrate toddlers’ dynamic looking after they heard the labels or cues (Twomey et al., 2017). Second, we reported toddlers’ proportion looking time to distractors to draw a full picture of their looking during retention.

Participants’ Proportion Target Looking in 100 ms Time bins

The time bin analysis was conducted in a 6500 ms time window after the first label or cue onset. The proportion target looking time of every 100ms time bin was compared with chance (.33) by one sample test, then a bootstrapped cluster-based permutation analysis (bootstrapped samples: 2000) was employed to examine the possibility of getting the statistically significant effect, the effect lasted for at least 200ms and the possibility (%) of getting significant effect were reported as references; a possibility of 15% threshold was suggested as a reliable reference (Dink & Ferguson, 2015; Maris & Oostenveld, 2007; Wendt et al., 2014). Figure S1 and S2 presents toddlers’ proportion target looking time of every 100 ms time bin after hearing the labels and affective cues in two retention phases respectively.

**Label trials in RT1.** After the *neutral* object was labeled, toddlers did not look to neutral target but looked to distractors from 100 to 300 ms (56.45%) from the label onset, while they did not look to positive or negative distractor particularly. After *positive* object was labeled, toddlers’ looking to it around chance for the entire trial. After *negative* object was labeled, toddler looked to target in 3100-3500 ms (27.10%), 4000-4200 ms (48.75%), and 5600-6100 ms (15.60%).

**Label trials in RT2.** After the *neutral* object was labeled, toddlers looked to it in 900-1100 ms (50.05%), 1400-2100 ms (6.60%), 2400-2600ms (47.65%), and 3600-3800ms (39.90%). After *positive* object was labeled, toddlers looked to distractors in 400-600 ms (37.90%), while they looked to negative distractor in 200-500 ms (40.55%). After *negative* object was labeled, toddler looked to target in 1300-1600 ms (35.85%), 4200-4500 ms (35.10%), and 4900-5100 ms (46.60%).

*
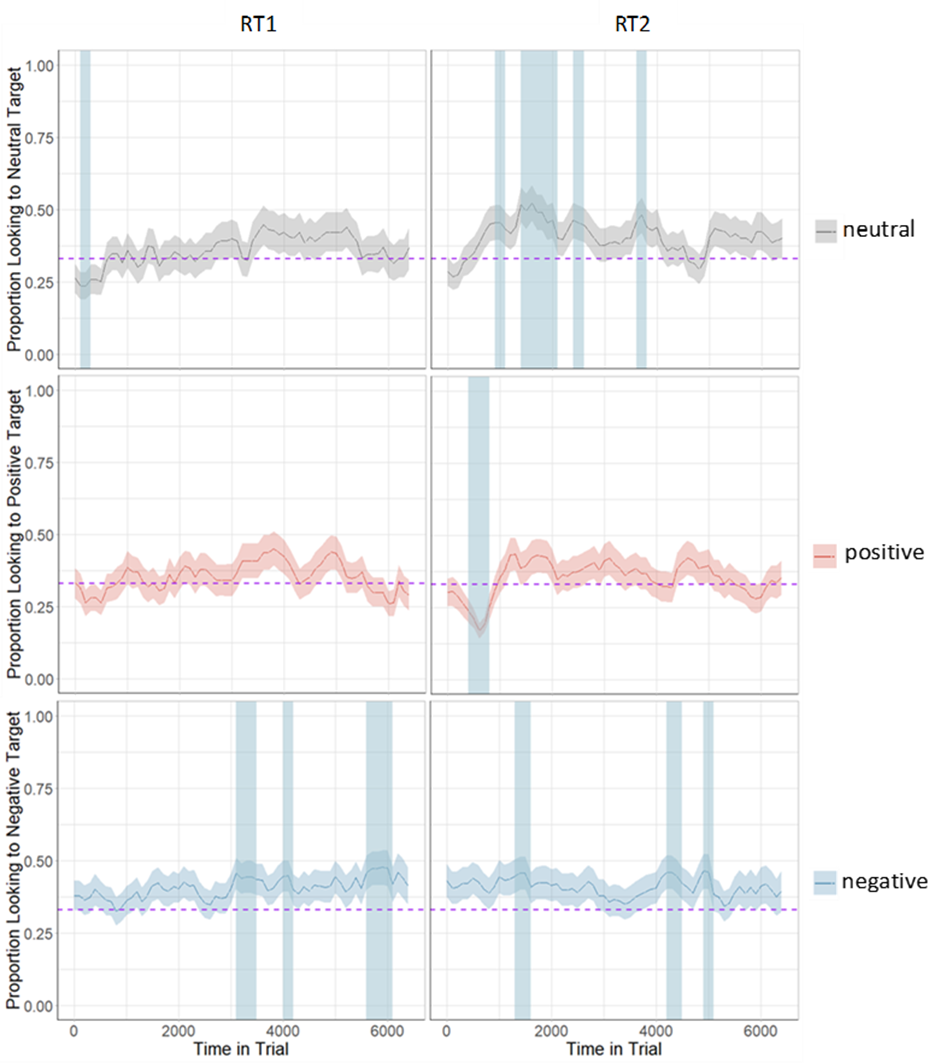
*

*Figure S1*. Toddlers’ Proportion Target Looking in Label Trials in Retention Phases. The labels onsets were at 0 ms, 2000 ms and 4000 ms respectively. The dashed line represents chance (.33). The light blue shadow represents the time bins during which proportion object looking time is different from the chance (*p* < .05).

**No-label trials in RT1.** After hearing the neutral cues *look*, toddlers did not look to neutral object but looked to distractors from 5200 to 5500 ms (25.85), they looked to the negative distractor in 3100-3300 ms (56.25%) and 5100-5800 ms (4.00%). After hearing the positive cues *wow*, toddlers’ looking to positive object around chance for the entire trial, but they looked to negative distractor in 4900-5100 ms (42.95%). After hearing the negative cues *ugh*, toddlers looked to negative target in 1100-4900 ms (0.00%) and 6000-6200 ms (39.80%).

**No-label trials in RT2.** After hearing the neutral cues *look*, toddlers did not look to neutral object but looked to distractors from 2300 to 2700 ms (20.80%), and 3000 to 3400 ms (22.50%) from cue onset, they looked to negative object in 1500-1700 ms (53.65%) and to positive object in 3400-3700 ms (25.25%) on the time course. After hearing the positive cues *wow*, toddlers’ looking to positive object around chance for the entire trial. After hearing the negative cues *ugh*, toddlers looked to negative target in 0-300 ms (30.55%), 1800-2500 ms (7.35%), 2900-3100 ms (36.45%), 3500-4300 ms (4.05%).

Overall, after hearing labels, toddlers looked to the neutral and negative targets apart from the positive one. After hearing affective cues, toddlers only looked to negative target after hearing negative cues.

*
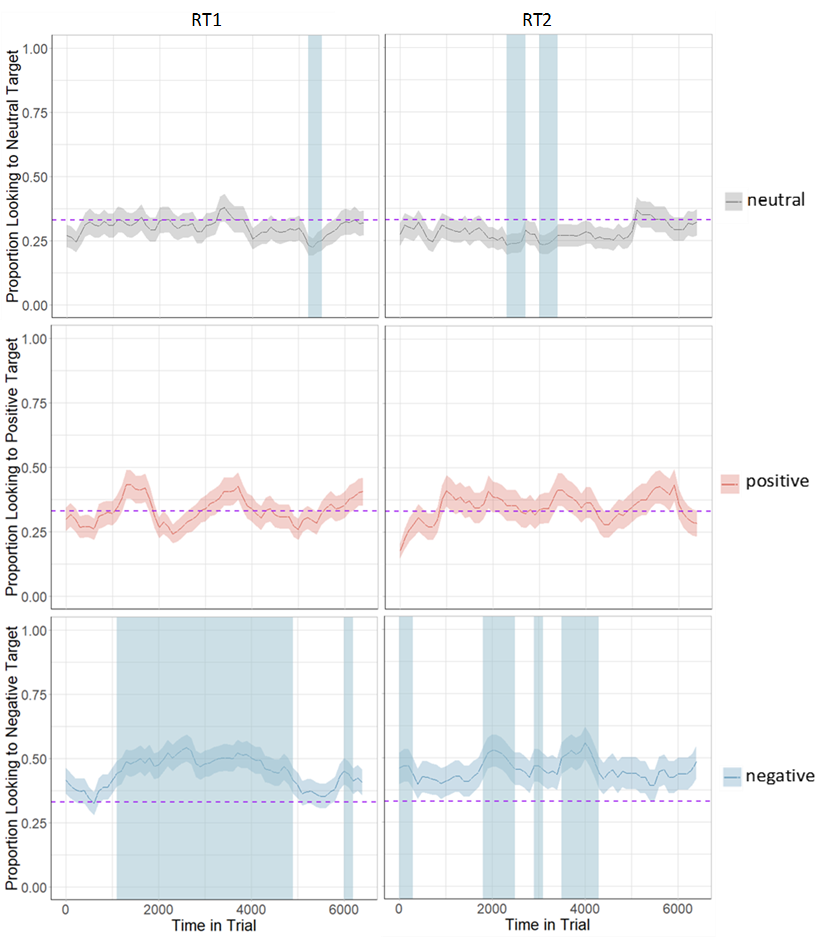
Figure S1*. Toddlers’ Proportion Target Looking in No-label Trials in Retention Phases. The cues onsets were at 0 ms, 2000 ms and 4000 ms (positive: 4200 ms) respectively. The dashed line represents chance (.33). The light blue shadow represents the time bins during which proportion object looking time is different from the chance (*p* < .05).

Participants’ Proportion Looking to Distractors

Toddlers’ looking to distractors in the retention phases was examined by comparing the proportion looking to distractor AOIs in a 6500 ms time window after the first label onset in both label and no-label trials against chance (.33) with a two-tailed, one-sample *t*-test.

**Label trials in RT1.** For neutral targets, toddlers’ looking to the negative distractor was below chance, *M* = 0.23, *SD* = 0.18, *t* (15) = -2.19, *p* = .04, *d* = 0.57; while their looking to the positive distractor was at chance (*M* = 0.34, *SD* = 0.30, *t* (15) = 0.22, *p* = .82, *d* = 0.04, BF_01_ = 3.83). For positive targets, toddlers’ looking to the neutral and negative distractors was at chance (neutral: *M* = 0.31, *SD* = 0.29, *t* (18) = -0.30, *p* = .77, *d* = -0.08, BF_01_ = 4.04; negative: *M* = 0.29, *SD* = 0.22, *t* (18) = -0.78, *p* = .45, *d* = 0.19, BF_01_ = 3.22). For negative targets, toddlers’ looking to the neutral distractor was at chance, *M* = 0.31, *SD* = 0.27, *t* (22) = -0.34, *p* = .73, *d* = -0.08, BF_01_ = 4.33; their looking to positive distractor was below chance, *M* = 0.19, *SD* = 0.18, *t* (22) = -3.68, *p* = .001, *d* = -0.78.

**Label trials in RT2.** For neutral targets, toddlers’ looking to negative distractors was below chance, *M* = 0.21, *SD* = 0.16, *t* (18) = -3.08, *p* = .006, *d* = 0.73; while their looking to positive distractors was at chance, *M* = 0.29, *SD* = 0.21, *t* (18) = -0.78, *p* = .44, *d* = 0.19, BF_01_ = 3.21. For positive targets, toddlers’ looking to neutral and negative distractors was at chance (neutral: *M* = 0.25, *SD* = 0.20, *t* (19) = -1.75., *p* = .10, *d* = .41, BF_01_ = 1.18; negative: *M* = 0.36, *SD* = 0.26, *t* (19) = 0.56, *p* = .59, *d* = .11, BF_01_ = 3.74). For negative targets, toddlers’ looking to the neutral distractor was at chance, *M* = 0.31, *SD* = 0.20, *t* (20) = -0.39, *p* = .70, *d* = -0.41, BF_01_ = 4.10; their looking to positive distractor was below chance, *M* = 0.17, *SD* = 0.16, *t* (20) = -4.56, *p* < .001, *d* = -1.02.

**No-label trials in RT1.** After hearing neutral cues, toddlers’ looking to the negative distractor was above chance, *M* = 0.45, *SD* = 0.17, *t* (21) = 3.32, *p* = .003, *d* = 0.69; while their looking to the positive distractor was at chance, *M* = 0.28, *SD* = 0.18, *t* (21) = -1.39, *p* = .18, *d* = 0.32, BF_01_ = 1.93. After hearing positive cues, toddlers’ looking to neutral and negative distractors was at chance (neutral: *M* = 0.31, *SD* = 0.17, *t* (22) = -0.61, *p* = .55, *d* = .15, BF_01_ = 3.87; negative: *M* = 0.35, *SD* = 0.18, *t* (22) = 0.67, *p* = .51, *d* = .12, BF_01_ = 3.74). After hearing negative cues, toddlers’ looking to neutral and positive distractors were below chance (neutral: *M* = 0.23, *SD* = 0.19, *t* (25) = -2.49, *p* = .02, *d* = -0.51; positive: *M* = 0.19, *SD* = 0.13, *t* (25) = -5.45, *p* < .001, *d* = -1.10).

**No-label trials in RT2.** After hearing neutral cues, toddlers’ looking to negative and positive distractors was at chance (positive: *M* = 0.33, *SD* = 0.18, *t* (21) = 0.09, *p* = .93, *d* = 0.001, BF_01_ = 4.47; negative: *M* = 0.43, *SD* = 0.25, *t* (21) = 1.92, *p* = .07, *d* = .40, BF_01_ = 0.95). After hearing positive cues, toddlers’ looking to neutral and negative distractor was at chance (neutral: *M* = 0.28, *SD* = 0.19, *t* (16) = -1.05, *p* = .31, *d* = .27, BF_01_ = 2.50; negative: *M* = 0.35, *SD* = 0.29, *t* (16) = 0.22, *p* = .83, *d* = .04, BF_01_ = 3.93). After hearing negative cues, toddlers’ looking to the neutral distractor was at chance (*M* = 0.26, *SD* = 0.25, *t* (17) = -1.26, *p* = .22, *d* = -0.31, BF_01_ = 2.08); their looking to the positive distractor was below chance (*M* = 0.15, *SD* = 0.19, *t* (17) = -3.95, *p* = .001, *d* = -0.95).

The Comparison of Target Looking between Two Retention phases cross Trial Types

A LMEM with the dependent variable of the proportion target looking time, the fix effects of an interaction of affect, retention phases (RT1, RT2) and trial types (label and no-label trials), and the random intercepts for items, participants and affect (*χ^2^*(1) = 13.03, *p* < .001) revealed a significant results, *χ^2^*(11) = 47.43, *p* < .001, *R^2^_m_* = .16, *R^2^_c_* = .17. However, a planned post-hoc Tukey’s HSD tests indicated that the proportion target looking time was not different cross trial types and cross two retention phases based on affect.

When *neutral* object was targeted, the proportion target looking time was not different between the retention phase based on the trial types (label: *β* = 0.07, *SE* = 0.09, *z* = 0.79, *p* = 1.00, BF_01_ = 2.23; no-label: *β* = -0.04, *SE* = 0.08, *z* = -0.48, *p* = 1.00, BF_01_ = 2.88). Meanwhile, the proportion target looking time was not different between label and no-label trials within the same retention phases (RT1: *β* = 0.15, *SE* = 0.08, *z* = 1.81, *p* = 0.81, BF_01_ = 0.64; RT2: *β* = 0.26, *SE* = 0.08, *z* = 3.22, *p* = .06, BF_01_ = 0.06), or between the retention phases (no-label.RT2-label.RT1: *β* = -0.19, *SE* = 0.08, *z* = -2.25, *p* = 0.51, BF_01_ = 0.48; label.RT2-nolabel.RT1: *β* = 0.22, *SE* = 0.08, *z* = 2.75, *p* = 0.20, BF_01_ = 0.07). However, the Bayes factors^[[1]](#footnote-1)^ revealed that the proportion target looking time in label trials in RT2 was higher than that in no-label trials in RT1 and RT2, suggesting that toddlers might sustained their looking to the neutral target after hearing the labels for longer time than hearing the neutral cues, and this effect was stronger overnight than only 5-minutes after referent selection phase.

When *positive* object was targeted, the proportion target looking time was not different between the retention phase based on the trial types (label: *β* = -0.01, *SE* = 0.08, *z* = -0.17, *p* = 1.00, BF_01_ = 3.17; no-label: *β* = 0.04., *SE* = 0.08, *z* = 0.46, *p* = 1.00, BF_01_ = 2.89). Meanwhile, the proportion target looking time was not different between label and no-label trials within the same retention phases (RT1: *β* = 0.06, *SE* = 0.08, *z* = 0.79, *p* = 1.00, BF_01_ = 2.50; RT2: *β* = 0.01, *SE* = 0.08, *z* = 0.14, *p* = 1.00, BF_01_ = 3.11), or between the retention phases (no-label.RT2-label.RT1: *β* = -0.03, *SE* = 0.09, *z* = -0.29, *p* = 1.00, BF_01_ = 3.01; label.RT2-nolabel.RT1: *β* = 0.05, *SE* = 0.08, *z* = 0.63, *p* = 1.00, BF_01_ = 2.69)

When *negative* object was targeted, the proportion target looking time was not different between the retention phase based on the trial types (label: *β* = 0.03, *SE* = 0.07, *z* = 0.34, *p* = 1.00, BF_01_ = 3.25; no-label: *β* = 0.01, *SE* = 0.08, *z* = 0.16, *p* = 1.00, BF_01_ = 3.29). Meanwhile, the proportion target looking time was not different between label and no-label trials within the same retention phases (RT1: *β* = -0.08, *SE* = 0.07, *z* = -1.11, *p* = 0.99, BF_01_ = 2.21; RT2: *β* = -0.07, *SE* = 0.08, *z* = -0.82, *p* = 1.00, BF_01_ = 2.74), or between the retention phases (no-label.RT2-label.RT1: *β* = 0.09, *SE* = 0.08, *z* = 1.16, *p* = 0.99, BF_01_ = 2.21; label.RT2-nolabel.RT1: *β* = -0.05, *SE* = 0.07, *z* = -0.73, *p* = 1.00, BF_01_ = 2.91).

Overall, despite the possibility that toddlers might sustained their looking to the neutral target longer by hearing labels on the second day than hearing cues on both days, their looking to objects was not different between label and no-label trials as well as time of testing when objects were associated with positive and negative affect.

The Effect of Vocabulary Size and Age on Participants’ Retention

Additionally, we submitted proportion target looking time to LMEMs with fixed effects of age and productive vocabulary to examine whether vocabulary size and age influenced toddlers’ target looking in the label and no-label trials in both RT phases. For the label trials, LMEMs with random intercepts for items and participants revealed no evidence for an effect of toddlers’ productive vocabulary (*χ*^2^(1) = 3.22, *p* = .07, *β* = 0.0009, *SE* = 0.0005, *t* = 1.79, *p* = .09, BF_01_ = 2.90) or in RT2 (*χ*^2^(1) = 0.02, *p* = .89, *β* = 0.0001, *SE* = 0.0008, *t* = 0.14, *p* = .89, BF_01_ = 3.61). We found evidence for an effect of age in RT1, with toddlers’ looking to targets decreasing as age increased (*χ*^2^(1) = 4.86, *p* = .03, *β* = -0.004, *SE* = 0.0018, *t* = -2.20, *p* = .04); but there was no evidence for an effect of age in RT2 (*χ*^2^(1) = 0.02, *p* = .88, *β* = 0.0003, *SE* = 0.002, *t* = 0.15, *p* = .99, BF_01_ = 3.66).

For the no-label trials, a LMEM with random intercepts for items and participants revealed no evidence for an effect of productive vocabulary in RT1 (*χ*^2^(1) = 0.18, *p* = .67, *β* = -0.0002, *SE* = 0.0005, *t* = -0.43, *p* = .67, BF_01_ = 2.67); nor an effect of age in RT1 (*β* = -0.0001, *SE* = 0.001, *t* = -0.11, *p* = 0.92, BF_01_ = 3.22). A LMEM with random intercepts for items, participants and affect (*χ*^2^(1) = 5.27, *p* = .02) revealed no evidence for an effect of productive vocabulary in RT2 (*χ*^2^(1) = 0.31, *p* = .58, *β* = -0.0005, *SE* = 0.0009, *t* = -0.56, *p* = 0.58, BF_01_ = 3.37); but there was evidence for an effect of age in RT2, with proportion target looking increasing as age increased (*χ*^2^(1) = 4.42, *p* = .04, *β* = 0.004, *SE* = 0.002, *t* = 2.10, *p* = .04).

Overall, toddlers’ productive vocabulary showed no effect on looking to the targets, but toddlers’ age related to proportion target looking negatively in the label trials in RT1 but positively in the no-label trials in RT2.

Experiment 2

Participants’ Looking Behaviors in Retention Phases

First, due to the possibility that the salience of negative object might affect toddlers’ looking, we explored toddlers’ proportion target looking in *neutral-negative* object pair in the label trials by the time bin analyses. Second, we compared proportion target looking time in the label trials between retention phases cross the object pairs.

Time Bin Analyses

To explore toddlers’ proportion target looking in *neutral-negative* object pair in the label trials. We compared their looking to neutral target with chance (.50) and between two retention phases. The time bin analysis was conducted in a 6500 ms time window after the first label onset. The proportion target looking time of every 100ms time bin was compared with chance by one sample test and between two retention phases by pairwise t-test, then a bootstrapped cluster-based permutation analysis (bootstrapped samples: 2000) was employed to examine the possibility of getting the statistically significant effect, the effect lasted for at least 200ms and the possibility (%) of getting significant effect were reported as references; a possibility of 15% threshold was suggested as a reliable reference (Dink & Ferguson, 2015; Maris & Oostenveld, 2007; Wendt et al., 2014).

**Neutral object as target in *neutral-negative* pair.** In RT1, toddlers looked to neutral target from 1300 to 1800 ms (17.10%) after hearing the label of neutral object. In RT2, toddlers looked to neutral target in 2100-3100 ms (1.80%), 3300-3600 ms (38.35%), and 6300-6500 ms (43.45%) after hearing the labels. The comparison of toddlers’ proportion neutral target looking time between two retention phases revealed that toddlers looked more to neutral target from 2500 to 2700 ms (62.00%) in RT2 (Figure S3). The results revealed that toddlers looked more to neutral targets on the second day, suggesting their looking to task-relevant object was less influenced by the negative distractor overnight.


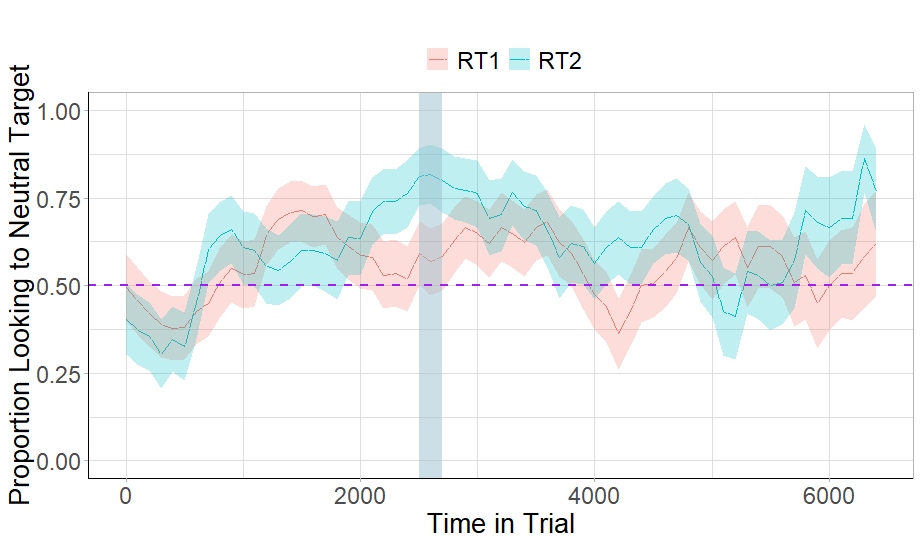
*Figure S3*. Comparison of Toddlers’ Proportion Neutral Target Looking in *neutral-negative* pair in Label Trials between Two Retention Phases. The labels onsets were at 0 ms, 2000 ms and 4000 ms respectively. The dashed line represents chance (.50). The light blue shadow represents the time bins during which proportion object looking time is different between two retention phases (*p* < .05).

The Comparison of Target Looking between Two Retention Phases in Label Trials

A LMEM with the dependent variable of the proportion target looking time in the label trials, fixed effect of an interaction of affect, object pairs (*neutral-positive*, *neutral-negative*, *negative-positive*) and RT phases (RT1, RT2), and random intercepts for items, participants and affect (*χ^2^*(1) = 4.27, *p* = .04) revealed a significant results, *χ^2^*(11) = 21.96, *p* = .02, *R^2^_m_* = .05, *R^2^*_c_ = .20. However, a planned post-hoc Tukey’s HSD tests indicated that the proportion target looking time was not different cross object pairs between two retention phases based on affect.

When *neutral* object was labeled, the proportion target looking time was not different between two retention phases for the *neutral-positive* (*β* = 0.02, *SE* = 0.05, *z* = 0.45, *p* = 1.00, BF_01_ = 3.60) and *neutral-negative* pairs (*β* = 0.05., *SE* = 0.05, *z* = 0.84, *p* = 1.00, BF_01_ = 3.00). Meanwhile, the proportion target looking time was not different cross object pairs between two retention phases (*neutral-positive*.RT2- *neutral-negative.*RT1: *β* = 0.08, *SE* = 0.05, *z* = 1.58, *p* = 0.92, BF_01_ = 1.99; *neutral-negative.*RT2-*neutral-positive*.RT1: *β* = -0.01, *SE* = 0.05, *z* = -0.23, *p* = 1.00, BF_01_ = 3.50).

When *positive* object was labeled, the proportion target looking time was not different between two retention phases for the *neutral-positive* (*β* = 0.11, *SE* = 0.06, *z* = 1.90, *p* = 0.76, BF_01_ = 0.95) and *negative-positive* pairs (*β* = 0.04, *SE* = 0.05, *z* = 0.77, *p* = 1.00, BF_01_ = 2.67). Meanwhile, the proportion target looking time was not different cross object pairs between two retention phases (*neutral-positive*.RT2-*negative-positive.*RT1: *β* = 0.07, *SE* = 0.06, *z* = 1.13, *p* = 0.99, BF_01_ = 1.99; *negative-positive.*RT2-*neutral-positive*.RT1: *β* = 0.09, *SE* = 0.05, *z* = 1.61, *p* = 0.91, BF_01_ = 1.05).

When *negative* object was labeled, the proportion target looking time was not different between two retention phases for the *neutral-negative* (*β* = 0.09, *SE* = 0.05, *z* = 1.64, *p* = 0.89, BF_01_ = 1.53) and *negative-positive* pairs (*β* = 0.04, *SE* = 0.06, *z* = 0.65, *p* = 1.00, BF_01_ = 3.26). Meanwhile, the proportion target looking time was not different cross object pairs between two retention phases (*neutral-negative*.RT2-*negative-positive.*RT1: *β* = 0.09, *SE* = 0.05, *z* = 1.76, *p* = 0.84, BF_01_ = 1.67; *negative-positive.*RT2-*neutral-negative*.RT1: *β* = 0.03, *SE* = 0.05, *z* = 0.49, *p* = 1.00, BF_01_ = 3.38).

Overall, toddlers’ looking to target label-object associations was not influenced by the affect associated with distractors and by the timing of testing.

The Effect of Vocabulary Size and Age on Participants’ Retention

LMEM with fixed effects of age and productive vocabulary and random intercepts for items and participants revealed no evidence for an effect of toddlers’ productive vocabulary in RT1 (*χ*^2^(1) = 0.09, *p* = .76, *β* = 0.0001, *SE* = 0.0004, *t* = 0.30, *p* = .72, BF_01_ = 4.28) or in RT2 (*χ*^2^(1) = 0.005, *p* = .95, *β* = 0.00002, *SE* = 0.0004, *t* = 0.07, *p* = .95, BF_01_ = 5.44). No evidence for an effect of age was found in RT1 (*χ*^2^(1) = 1.56, *p* = .21, *β* = -0.001, *SE* = 0.0009, *t* = -1.25, *p* = .22, BF_01_ = 1.97) or in n RT2 (*χ*^2^(1) = 1.97, *p* = .16, *β* = -0.0001, *SE* = 0.0001, *t* = -1.40, *p* = .17, BF_01_ = 1.62). Overall, toddlers’ productive vocabulary and age showed no effect on looking to the targets in the label trials in Experiment 2.

References

Dink, J. W., & Ferguson, B. (2015). *eyetrackingR: An R library for eye-tracking data analysis. Retrieved from http://www.eyetracking-r.com.*

Koolagudi, S. G., Ray, S., & Sreenivasa Rao, K. (2010). *Emotion classification based on speaking rate.* International Conference on Contemporary Computing, Noida, India.

Maris, E., & Oostenveld, R. (2007). Nonparametric statistical testing of EEG- and MEG-data. *Journal of Neuroscience Methods*, *164*(1), 177-190. https://doi.org/10.1016/j.jneumeth.2007.03.024

Twomey, K. E., Ma, L., & Westermann, G. (2017). All the right noises: background variability helps early word learning. *Cognitive Science*. https://doi.org/10.1111/cogs.12539

Wendt, D., Brand , T., & Kollmeier, B. (2014). An eye-tracking paradigm for analyzing the processing time of sentences with different linguistic complexities. *PLoS ONE*, *9*(6), e100186. https://doi.org/10.1371/journal.pone.0100186

Wiley, J., & Jarosz, A. F. (2014). What are the odds? A practical guide to computing and reporting bayes factors. *The Journal of Problem Solving*, *7*(1). https://doi.org/10.7771/1932-6246.1167

1. When BF_01_ is smaller than 0.1, the insignificant frequentist analyses was considered supportive for the alternative hypotheses, that is, it indicates a strong evidence that the experimental variables had effect on the dependent variables (for deail description, see Wiley et al., 2014). [↑](#footnote-ref-1)
